# Sevoflurane Exposure Induces Neuronal Cell Ferroptosis Initiated by Increase of Intracellular Hydrogen Peroxide in the Developing Brain via ER Stress ATF3 Activation

**DOI:** 10.1007/s12035-023-03695-z

**Published:** 2023-10-24

**Authors:** Liheng Kang, Meihua Piao, Nan Liu, Wanping Gu, Chunsheng Feng

**Affiliations:** Department of Anesthesiology, The First Hospital of Jilin University, No. 1 Xinmin St., Changchun, 130021 China

**Keywords:** Sevoflurane, Ferroptosis, Hydrogen peroxide, ER stress, ATF3, Developing brain

## Abstract

Neuronal cell death is acknowledged as the primary pathological basis underlying developmental neurotoxicity in response to sevoflurane exposure, but the exact mechanism remains unclear. Ferroptosis is a form of programmed cell death characterized by iron-dependent lipid peroxidation that is driven by hydrogen peroxide (H_2_O_2_) and ferrous iron through the Fenton reaction and participates in the pathogenesis of multiple neurological diseases. As stress response factor, activating transcription factor 3 (ATF3) can be activated by the PERK/ATF4 pathway during endoplasmic reticulum (ER) stress, followed by increased intracellular H_2_O_2_, which is involved in regulation of apoptosis, autophagy, and ferroptosis. Here, we investigated whether ferroptosis and ATF3 activation were implicated in sevoflurane-induced neuronal cell death in the developing brain. The results showed that sevoflurane exposure induced neuronal death as a result of iron-dependent lipid peroxidation damage secondary to H_2_O_2_ accumulation and ferrous iron increase, which was consistent with the criteria for ferroptosis. Furthermore, we observed that increases in iron and H_2_O_2_ induced by sevoflurane exposure were associated with the upregulation and nuclear translocation of ATF3 in response to ER stress. Knockdown of ATF3 expression alleviated iron-dependent lipid peroxidation, which prevented sevoflurane-induced neuronal ferroptosis. Mechanistically, ATF3 promoted sevoflurane-induced H_2_O_2_ accumulation by activating NOX4 and suppressing catalase, GPX4, and SLC7A11 expression. Additionally, an increase in H_2_O_2_ was accompanied by the upregulation of TFR and TF and downregulation of FPN, which linked iron overload to ferroptosis induced by sevoflurane. Taken together, our results demonstrated that ER stress-mediated ATF3 activation contributed to sevoflurane-induced neuronal ferroptosis via H_2_O_2_ accumulation and the resultant iron overload.

**Supplementary Information:**

The online version contains supplementary material available at 10.1007/s12035-023-03695-z.

## Introduction

The safety of volatile anesthetics in neonates and infants has attracted extensive attention from anesthesiologists because of potential neurotoxicity in the developing brain [1, 2]. Accumulating evidence has proven that sevoflurane, a volatile anesthetic that is extensively used in pediatric practice, may induce widespread neurodegeneration and persistent cognitive impairment in a variety of neonatal animal models, and these effects are exacerbated following multiple rounds or prolonged anesthesia [3–5]. Clinical studies have indicated that exposure to sevoflurane throughout a vital period of human brain growth is linked with an increased risk of aberrant neurobehavioral alterations in childhood [6–8]. Although increasing evidence has demonstrated that neuronal cell death is one of the primary pathological bases underlying developmental neurotoxicity caused by volatile anesthetics and multiple studies have proposed that sevoflurane can trigger neuronal cell death through the induction of apoptosis and Parthanatos in the developing brain [9–11], the exact mechanism of neonatal neuronal death after sevoflurane exposure remains largely unknown.

Ferroptosis is a form of programmed cell death that is characterized by an increase in intracellular ferrous iron and resulting lipid peroxidation, which results in lethal damage to polyunsaturated fatty acids (PUFAs), ultimately leading to cell membrane injury and cell death [12–15]. Studies have linked ferroptosis to the pathogenesis of a variety of neurological diseases, such as cerebral ischemia and anoxia, stroke, and traumatic brain injury [16–18]. Remarkably, the immature brain of infants contains more iron and PUFAs and has high oxygen uptake and low levels of antioxidants, which make it particularly sensitive to the consequences of iron-dependent lipid peroxidation [19, 20]. In addition, a recent study demonstrated that sevoflurane exposure could induce iron metabolic abnormalities in neonatal mice, which were accompanied by behavioral and cognitive impairment [21]. However, whether ferroptosis is implicated in neuronal death following sevoflurane exposure in the developing brain remains to be determined.

Hydrogen peroxide (H_2_O_2_) plays a crucial role in the pathogenesis of ferroptosis, and it not only reacts with ferrous iron through the Fenton reaction to generate hydroxyl radicals, which cause high levels of lipid peroxidation damage to PUFAs [22], but also promotes the upregulation of transferrin receptor (TFR) [23]. The endoplasmic reticulum, which is an essential site associated with ferroptosis, has been reported to be a central hub that drives the metabolism of many lipids and PUFAs and can promote lipid peroxidation and cell death [24, 25]. Moreover, endoplasmic reticulum (ER) stress improves intracellular H_2_O_2_ levels and participates in the regulation of various nervous system diseases and ischemic injury [26–28]. As a signal downstream of the ER stress PERK/ATF4 pathway, activating transcription factor 3 (ATF3), a stress response factor that belongs to the ATF/CREB transcription factor family, is overactivated during ER stress, followed by excess H_2_O_2_ accumulation and oxidative stress damage, which is responsible for different types of cell death, such as apoptosis and autophagic cell death [29–31]. Currently, several studies have demonstrated that ATF3 overexpression contributes to the increases in H_2_O_2_ and iron, which mediate ferroptosis in retinal pigment epithelial cells and glioma cells [28, 31, 32]. In addition, excessive ER stress and the resultant cell death have been well established as etiological factors in developmental neurotoxicity after sevoflurane exposure [33]. Additionally, emerging evidence showed that exposure to sevoflurane could result in neuronal cell death and neurobehavioral impairment, which were associated with increased intracellular H_2_O_2_ and lipid peroxidation [13, 33, 34]. Thus, sevoflurane-induced neuronal death is accompanied by ER stress, which links ATF3 activation to ferroptosis induced by H_2_O_2_ and iron overload. In light of these studies, we hypothesized that sevoflurane induced ER stress and ATF3 activation, which caused excessive intracellular H_2_O_2_ accumulation, thereby resulting in an increase in ferrous iron and subsequently leading to neuronal cell ferroptosis in the developing brain.

Thus, in this study, we determined whether ferroptosis and ATF3 were involved in neuronal cell death following sevoflurane exposure by examining neuronal cells in vitro and neonatal ATF3-knockout mice in vivo and then examined the role of ER stress and ATF3-induced H_2_O_2_ accumulation in the regulation of iron-dependent neuronal cell death induced by sevoflurane in the developing brain.

## Materials and Methods

### Primary Reagents and Antibodies

Maruishi Pharmaceutical Co. (Osaka, Japan) provided the sevoflurane. Liproxstatin-1 (Lip-1), hydrogen peroxide (H_2_O_2_), ferrostatin-1 (Fer-1), N-acetyl-L-cysteine (NAC), deferoxamine (DFO), and glutathione (GSH) were acquired from Sigma‒Aldrich Company (St. Louis, USA). GKT137831, GSK2606414, and deferiprone (DFP) were acquired from Selleck Chemicals (Houston, USA). Primary antibodies against ATF4 (ab216839), GPX4 (ab125066), ATF3 (ab207434), GRP78 (ab21685), catalase (ab209211), amino acid antiporter System Xc − (SLC7A11) (ab175186), ferritin light chain (FTL) (ab69090), ferritin heavy chain (FTH) (EPR18878), FPN (ab235166), TF (ab82411), and TFR (ab84036) were obtained from Abcam (Cambridge, UK). Antibodies against NOX4 (NB110-58851SS) were provided by Novus Biologicals (Littleton, CO, USA). Cell Signaling Technology (Beverly, USA) provided anti-PERK (#3192). All other reagents were obtained from Sigma or were the highest grade available.

### Cell Cultures and treatments

Mouse hippocampal HT22 cells were obtained from the Chinese Academy of Sciences (Shanghai, China). HT22 cells were grown in DMEM supplemented with 10% fetal bovine serum. Rat primary hippocampal neurons were collected from neonatal Sprague‒Dawley rats according to a previously reported method [35]. A 6-well culture plate coated with poly-D-lysine was used to seed hippocampal neurons, which were then cultured in neurobasal medium (Gibco, USA) that was supplemented with 2% B27. Experiments were conducted on primary hippocampal neurons after one week in vitro. Neuronal cells were grown in a humidified environment with 95% air and 5% CO_2_ at 37 °C. For in vitro studies, all neuronal cells received with or without sevoflurane in a gas mixture of 5% CO_2_, 21% O_2_, and balanced N_2_ at 37℃ in a tightly sealed plastic chamber (Billups-Rothenberg, USA) inside a cell culture incubator. The humidified gas mixture went through an agent-specific vaporizer at a flow rate of 2 L/min for 5 min and 0.5 L/min for the remaining exposure time. The neuronal cells were exposed sevoflurane at concentrations of 0, 2, 4, and 8% for 6, 12, and 24 h, respectively. An infrared probe (Ohmeda 5330) was used to constantly measure sevoflurane, oxygen, and carbon dioxide oxygen levels in the effluent gas throughout the experiment.

### Animals and Experimental Protocol

The First Hospital of Jilin University’s Ethics Committee authorized the use of animals in this study, which was conducted in accordance with the National Institutes of Health’s instructions for the care and use of laboratory animals. B6/JGpt-ATF3^em10Cd10705^/Gpt mice (ATF3^−/−^ mice) were obtained from GemPharmatech LLC. (Nanjing, China). Beijing Vital River Laboratory Animal Technology Co., Ltd. (China) provided 6-day-old C57BL/6 J mice. All neonatal mice were placed with their mothers under conventional laboratory settings (22 ± 1 °C, cycle of 12/12-h light–dark, 60% humidity atmosphere) with free access to food and water. There were several measures used to lessen the number of animals used and their pain.

For the experimental treatments, one hundred eighty postnatal day (P) 6 wild-type (WT) mice from thirty litters (each litter had 5–8 pups) of both sexes were randomly allocated into the following six groups (*n* = 30 per group): WT, WT + DFP, WT + GSK2606414, WT + 3% sevoflurane, WT + DFP + 3% sevoflurane, and WT + GSK2606414 + 3% sevoflurane. Sixty P6 ATF3-knockout (KO) mice of both sexes from ten litters (each litter consisted of 5–8 pups) were randomly divided into two groups: ATF3 KO mice and ATF3 KO + 3% sevoflurane (*n* = 30 per group). To treat the pups, GSK2606414 (50 mg/kg) or DFP (75 mg/kg) was intraperitoneally administered in approximately 0.1 mL 60 min prior to exposure to sevoflurane each day. From P6 to P8, the pups were anesthetized for 2 h each day in a chamber with 3% sevoflurane, as previously reported [36, 37]. Anesthesia was administered with 5% sevoflurane in 60% oxygen until the righting reflex disappeared and was maintained with 3% sevoflurane in 60% oxygen at a flow rate of 1 L/min for 2 h. Pups without sevoflurane anesthesia simply received the same oxygen levels and flow rates in an identical chamber. A warming pad was placed under the chamber for constant rectal temperature monitoring to maintain normothermia at 37 ± 0.5 °C throughout the experiment. Six neonatal mice from each group were euthanized with 8% sevoflurane in 60% oxygen at the end of anesthesia on P8 [38], and direct thoracotomy was conducted to remove a left ventricular arterial blood sample, which was immediately analyzed by a blood gas analyzer (ITC, Edison, NJ). The remaining neonatal mice were resuscitated with 60% oxygen at a flow rate of 3 L/min until the positive force reflex returned and then were returned to their home cages. In the present study, the pups were all alive following sevoflurane exposure.

### Cellular Viability and Cell Death Analysis

Formazan levels were measured with methyl thiazolyl tetrazolium (MTT) (Sigma–Aldrich, MO, USA), and the absorbance level at 570 nm was measured to assess cellular viability. A lactate dehydrogenase (LDH) cytotoxicity assay kit was used to evaluate cell death (Beyotime, China). The absorbance of each sample was measured at 490 nm according to the manufacturer’s instructions, and the cell death ratio was computed.

### Measurement of Intracellular Ferrous Iron

The iron colorimetric test kit obtained from Biovision (Milpitas, CA, USA) was used to determine the amount of ferrous iron. Briefly, the recovered neuronal cells and hippocampal tissues (10 mg) were processed by being mixed with iron assay buffer, homogenized on ice, and then centrifuged for 10 min at 16,000 g at 4 °C to obtain the supernatant. Iron standards and samples were incubated for 30 min at 37 °C. The sample absorbance was measured at 593 nm after 1 h of incubation in the dark at 37 °C with 100 μL of the iron probe. Ferrous iron levels were measured using a typical concentration curve. The outcomes are presented as a ratio to the level in control cells or tissues.

### Measurement of Glutathione, Malondialdehyde, Hydrogen Peroxide, and Cysteine

The test kits for glutathione (GSH), malondialdehyde (MDA), hydrogen peroxide (H_2_O_2_), and cysteine were purchased from the Nanjing Jiancheng Bioengineering Institute (Nanjing, China). According to the manufacturer’s guidelines, the corresponding samples and reagents were prepared and tested in strict accordance with the experimental procedure. The sample absorbance ratio to that of control tissues or cells was used to display the findings.

### Measurement of Intracellular Superoxide

The probe dihydroethidium (DHE) was used to detect superoxide levels (Beyotime Biotech, Nanjing, China). Six-well plates were used for cell culture. After being treated with 4% or 8% sevoflurane for 24 h, the cells were rinsed twice in PBS and then treated with DHE (10 μM) in fresh medium in the dark for 30 min at 37 °C. Images of DHE-stained neuronal cells were obtained with a fluorescence microscope (Olympus X71, Japan).

### Cell Transfection

Primary hippocampal neurons and HT22 cells were seeded in a 6-well culture dish for 1 day for RNA interference research. According to the manufacturer’s guidelines, we transfected the siRNA using Lipofectamine 3000 (Invitrogen, USA). The siRNAs used to transfect HT22 cells were synthesized by GenePharma (Suzhou, China), and the sequences were as follows: 5′-ATGGCCGGCTATGGATGAT-3′ for ATF4; 5′-GCGGCGAGAAAGAAAUAAATT-3′ for ATF3; and 5′-UUCUCCGAACGUGUCACGUTT-3′ for scrambled siRNA (negative control). The siRNAs used to transfect rat primary hippocampal neurons were synthesized by GenePharma, and the sequences were as follows: 5′-CCTCACTGGCGAGTGTAAA-3′ for ATF4; 5′-GCCGAAACAAGAAGAAGGATT-3′ for ATF3; and 5′-ACGUGACACGUUCGGAGAATT-3′ for scrambled siRNA (negative control). After 24 h of interference, neuronal cells were exposed to 8% sevoflurane for 24 h, and Western blotting was used to examine target gene knockdown efficiency. To explore the effects of GPX4 and SLC7A11 overexpression on ferroptosis, the vector or GPX4 and SLC7A11 plasmids (Hanbio Biotechnology, China) were transfected into HT22 cells in the presence of Lipofectamine 3000 (Invitrogen, USA) according to the manufacturer’s instructions. Following transfection for 48 h, HT22 cells were digested and cultured in 6- or 96-well plates. Then, HT22 cells were exposed to 8% sevoflurane for 24 h.

### Brain Tissue Harvesting

Brain samples were harvested after the pups were euthanized with 8% sevoflurane [38]. For the purposes of Western blotting and subsequent assays, ten pups per group were transcardially perfused with ice-cold PBS 6 h after sevoflurane exposure on P8, and the brain was promptly harvested to obtain the hippocampus. For hematoxylin and eosin staining, after repeated exposure to sevoflurane on day 7, six mice per group were fixed with 4% paraformaldehyde after transcardial perfusion of ice-cold PBS, and then the brains were removed and postfixed. The neonatal mice were perfused by an experienced researcher.

### Hematoxylin and Eosin (HE) Staining

Prior to hematoxylin staining, the brain samples were sliced to a thickness of 4 µm, deparaffinized, and submerged in distilled water. The samples were then thoroughly cleaned with distilled water, progressively dehydrated using an alcohol gradient, stained with eosin, dried and vitrified once more, and finally coated in resin. An observer who was blind to the experiment used a light microscope (Carl Zeiss, Germany) to examine the pyramidal neurons of the hippocampal CA1 area in each slide. The following criteria were used to determine neuronal death and damage: sparse and disorderly neuronal organization, cell shrinkage, cytoplasm that was morphologically pink, and pyknotic nuclei.

### Immunocytochemical Staining

HT22 cells were plated in a culture dish, treated with or without sevoflurane, fixed in ethanol, rinsed with phosphate buffered saline, and treated for 10 min with 1% Triton X-100. The neuronal cells were treated with anti-ATF3 (1:100) after the nonspecific antibody binding locations were blocked. Then, the samples were incubated for 1 h with Cy3-labeled goat anti-rabbit IgG (H + L) (1:500) and subsequently with Hoechst 33258. Finally, a researcher who was unaware of the group assignments observed the cells using a laser scanning confocal microscope (Olympus FV3000, Japan).

### Transmission Electron Microscopy

HT22 cells were treated with 8% sevoflurane for 24 h. Then, 0.25% trypsin was used to harvest the HT22 cells, after which the cells were rinsed with PBS and centrifuged at 2000 rpm for 10 min to collect the cells. After that, an ice-cold 2.5% glutaraldehyde solution was used to fix the cells in PBS, and the cells were washed with PBS, postfixed in a 1% osmium tetroxide solution with 0.1% potassium ferricyanide, dehydrated with various graduated ethanol concentrations, and immersed in Epon resin. Then, the samples were sliced, stained, and subsequently observed by transmission electron microscopy (JEOL, Pleasanton, USA).

### Western Blot Analysis

A nuclear and cytoplasmic protein extraction kit was used to isolate nuclear and cytoplasmic proteins. As previously stated, western blotting was performed [39]. The PVDF membranes were treated with 3% BSA in TBS for 30 min at room temperature after the proteins (20–30 µg) had been electrophoretically separated by SDS and transferred. The membranes were then incubated with primary antibodies overnight at 4 °C. The membranes were then incubated with the secondary antibody conjugated to horseradish peroxidase for 2 h at room temperature. The blots were visualized using an improved chemiluminescence developer, and ImageJ was used to conduct densitometry.

### Morris Water Maze Tests (MWM)

From postnatal day (P) 31 to P36, the spatial memory of mice that were treated with or without repeated sevoflurane administration from P6 to P8 was tested by using the MWM. The MWM test was performed as previously described [39]. In brief, the MWM is a basic steel pool with a diameter of 120 cm and a height of 60 cm, and it was filled with water to a depth of 1 cm over the top of a platform with a diameter of 10 cm. From one of four specified beginning points, the mice were gently dropped into the water with their noses against the wall. The duration required for the mice to travel to a sunken escape platform was used to assess spatial memory during the learning phase. Four learning experiments were performed each day for five consecutive days. The mice were given 60 s in each of these experiments to identify the hidden platform; if they were unable to do so, the trainer would lead them there, after which the mice were left on the platform for 15 s to learn. The average period for the mice to locate the hidden platform in the four daily experiments was measured as the escape latency. The mice swam freely for 60 s in the tank without the platform during the probe trial, which took place 24 h after the learning phase, and the duration spent in the initial platform quadrant was measured. A video tracking system was used for monitoring (Noldus Ltd., Etho Vision XT, Holland).

### Statistical Analysis

SPSS software (version 24.0) was used to conduct all statistical analyses (SPSS, IBM Corporation). The normality of the data was assessed using the Shapiro‒Wilk normality test. To compare statistical differences between two sets of data, a two-tailed unpaired *t* test was used. One-way and two-way ANOVA were used, and when needed, an LSD test was used to assess variations between more than two groups. To evaluate the behavioral data, a two-way analysis of variance (ANOVA) with repeated measures was used, followed by the Bonferroni multiple comparison test. The findings of the trial are shown as the mean ± SD. The evaluation used a *P* value of less than 0.05 to indicate significance.

## Results

### Sevoflurane Inhibited Cell Viability and Induced Neuronal Cell Death

To investigate the neurotoxicity of sevoflurane on neonatal neuronal cells, cellular viability was evaluated using the MTT assay after exposure of mouse hippocampal HT22 cells and rat primary hippocampal neurons to 2, 4, and 8% sevoflurane for 6, 12, and 24 h [10]. Mouse hippocampal HT22 cells are similar to undifferentiated neural cells and are particularly sensitive to oxidative stress. The primary hippocampal neurons of rats are immature neurons, which were isolated from neonatal Sprague‒Dawley rats. As shown in Supplementary Fig. 1a, sevoflurane exposure significantly reduced the viability of neuronal cells in a time- and concentration-dependent manner in contrast to those in the control group. Considering that the integrity of dying cell membranes was damaged, which produced a quantifiable amount of LDH, we used an LDH release test to determine whether sevoflurane induced neuronal cell death. Neuronal cells treated with 2% sevoflurane for 6 h showed an overt increase in cell death in contrast to cells in the control group, and this effect was exacerbated with increasing time and concentrations (Supplementary Fig. 1b). Consistently, microscopy showed that the majority of neuronal cells treated with 8% sevoflurane for 24 h were smaller in size and round in shape (Supplementary Fig. 1c). Thus, these findings demonstrated that exposure to sevoflurane reduced cell viability and resulted in neuronal cell death in a concentration- and time-dependent manner.

### Sevoflurane Induced Neonatal Neuronal Cell Ferroptosis

**Fig. 1:**
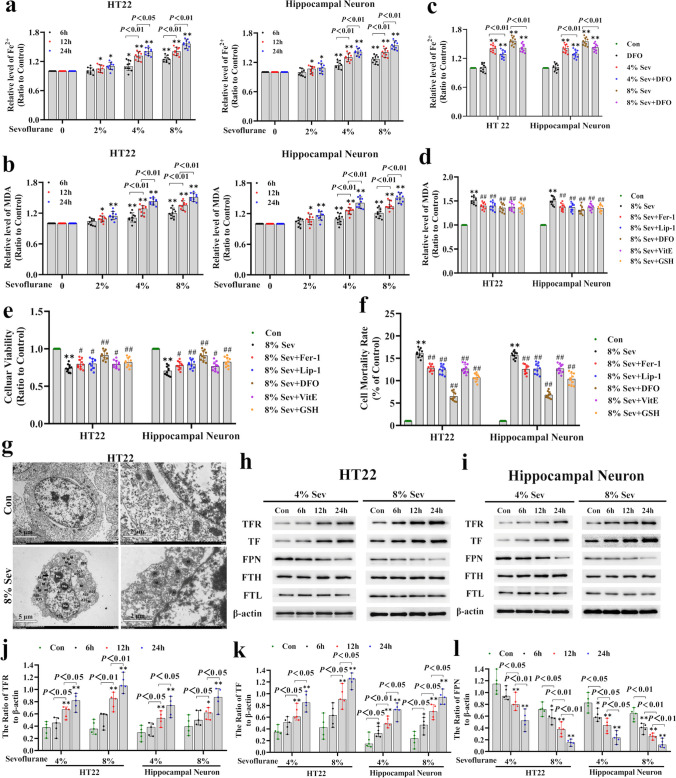
Sevofluraneinduced neonatal neuronal cell ferroptosis. (**a**) Iron assays revealed that sevoflurane increased intracellular ferrous iron in a concentration- and time-dependent manner. (**b**) The MDA assay showed that sevoflurane induced lipid peroxidation in a concentration- and time-dependent manner. (**c**) The iron assay demonstrated that DFO markedly suppressed the increase in intracellular iron in neonatal neuronal cells exposed to 4% or 8% sevoflurane for 24 h. (**d**) The MDA assay proved that lipid peroxidation caused by 8% sevoflurane exposure for 24 h in neuronal cells was alleviated in the presence of Fer-1 (50 μM), Lip-1 (10 μM), DFO (100 μM), VitE (100 μM), or GSH (2.5 mM). (**e**) The MTT assay proved that the reduction in neuronal cell viability induced by 8% sevoflurane for 24 h was significantly rescued by Fer-1, Lip-1, DFO, VitE, or GSH. (**f**) The LDH release assay revealed that neuronal cell death induced by 8% sevoflurane exposure for 24 h was markedly suppressed by Fer-1, Lip-1, DFO, VitE, or GSH. (**g**) Representative transmission electronic microscopy images showed that HT22 cells exposed to 8% sevoflurane for 24 h had irregularly shaped nuclei (N) with multiple nucleoli (Nu), a slightly widened perinuclear space (black arrows), shrunken mitochondria (M) with increased membrane density and expanded cristae, and local rough endoplasmic reticulum (RER) expansion compared to those in the control group. (**h–l**) Western blotting demonstrated that sevoflurane induced the upregulation of TFR and TF and downregulation of FPN in a time- and concentration-dependent manner, but there was no significant change in FTH or FTL. In comparison with the control group, ^∗^*p* < 0.05, ^∗∗^*p* < 0.01; compared with the group treated with sevoflurane, ^#^*p* < 0.05, ^##^*p* < 0.01

To elucidate the mechanism of sevoflurane-induced neonatal neuronal cell death, we determined whether ferroptosis was implicated in neuronal death caused by sevoflurane. Ferroptosis is typically characterized by increases in intracellular ferrous iron and lipid peroxidation [12], and we examined sevoflurane-induced changes in ferrous iron and the lipid peroxidation product MDA. We found that the levels of intracellular iron and MDA were increased when neuronal cells were subjected to 2% sevoflurane for 12 h in contrast to those in the control group, and these effects were further enhanced with increasing time and concentrations (Fig. 1a, b). These results suggested that sevoflurane could increase intracellular ferrous iron and lipid peroxidation in a time- and concentration-dependent manner in neuronal cells.

To verify whether ferrous iron induces lipid peroxidation and contributes to iron-dependent cell death, neuronal cells were administered the iron chelator deferoxamine (DFO) at a concentration of 100 μM 1 h prior to sevoflurane exposure. We found that inhibiting ferrous iron with DFO significantly suppressed sevoflurane-induced MDA production and the decrease in the viability of neuronal cells (Fig. 1c–e). Moreover, the LDH release test showed that sevoflurane-induced neuronal cell death was attenuated in the presence of DFO (Fig. 1f). Furthermore, DFO pretreatment restored the sevoflurane-induced reduction in neuronal cell size, as seen under a light microscope (Supplementary Fig. 1c). These results suggested that sevoflurane induced neonatal neuronal lipid peroxidation and cell death through an increase in intracellular ferrous iron. Then, to further investigate the involvement of lipid peroxidation in sevoflurane-induced neuronal cell death, neuronal cells were exposed to sevoflurane in the presence of the lipophilic antioxidants ferrostatin-1 (Fer-1, 50 μM) and liproxstatin-1 (Lip-1, 10 μM). We found that Fer-1 or Lip-1 pretreatment reversed the sevoflurane-induced increase in MDA and mitigated neonatal neuronal cell death induced by sevoflurane (Fig. 1d, f). Consistently, pretreatment with 100 μM vitamin E or 2.5 mM GSH markedly suppressed the sevoflurane-induced increase in MDA and attenuated the lethal effect of sevoflurane on neuronal cells (Fig. 1d, f). These results indicated that lipid peroxidation contributed to sevoflurane-induced neonatal neuronal cell death. Additionally, transmission electron microscopy revealed that HT22 cells exposed to 8% sevoflurane for 24 h had irregularly shaped nuclei (N) with multiple nucleoli (Nu), a slightly widened perinuclear space (black arrows), shrunken mitochondria (M) with increased membrane density and expanded cristae, and local rough endoplasmic reticulum (RER) expansion compared to those in the control group (Fig. 1g), which was consistent with the morphological features of ferroptosis [40]. Thus, sevoflurane exposure induced ferroptosis in neonatal neuronal cells.

To elucidate the mechanism of the sevoflurane-induced aberrant increase in intracellular iron, we examined the changes in proteins that could regulate intracellular iron metabolism [41]. In this study, we found that transferrin receptor (TFR), which mediates ferric iron uptake by cells through the iron-TF-TFR complex, was significantly upregulated after sevoflurane exposure in a time- and concentration-dependent manner (Fig. 1h–j). Correspondingly, the sevoflurane-induced expression of TF was increased, and this effect was exacerbated with increasing time and concentrations (Fig. 1h, i, k). Moreover, ferroportin (FPN), which mediates intracellular iron export, was markedly downregulated by each sevoflurane concentration and time, but there was no significant change in ferritin (both FTH and FTL), which could bind to intracellular ferrous iron after sevoflurane exposure (Fig. 1h, i, l). Thus, our data suggested that sevoflurane improved intracellular iron levels by upregulating TFR and TF and downregulating FPN but did not suppress ferritin (FT).

### H_2_O_2_ Was Implicated in Sevoflurane-Induced Neuronal Cell Ferroptosis via an Increase in Iron

**Fig. 2:**
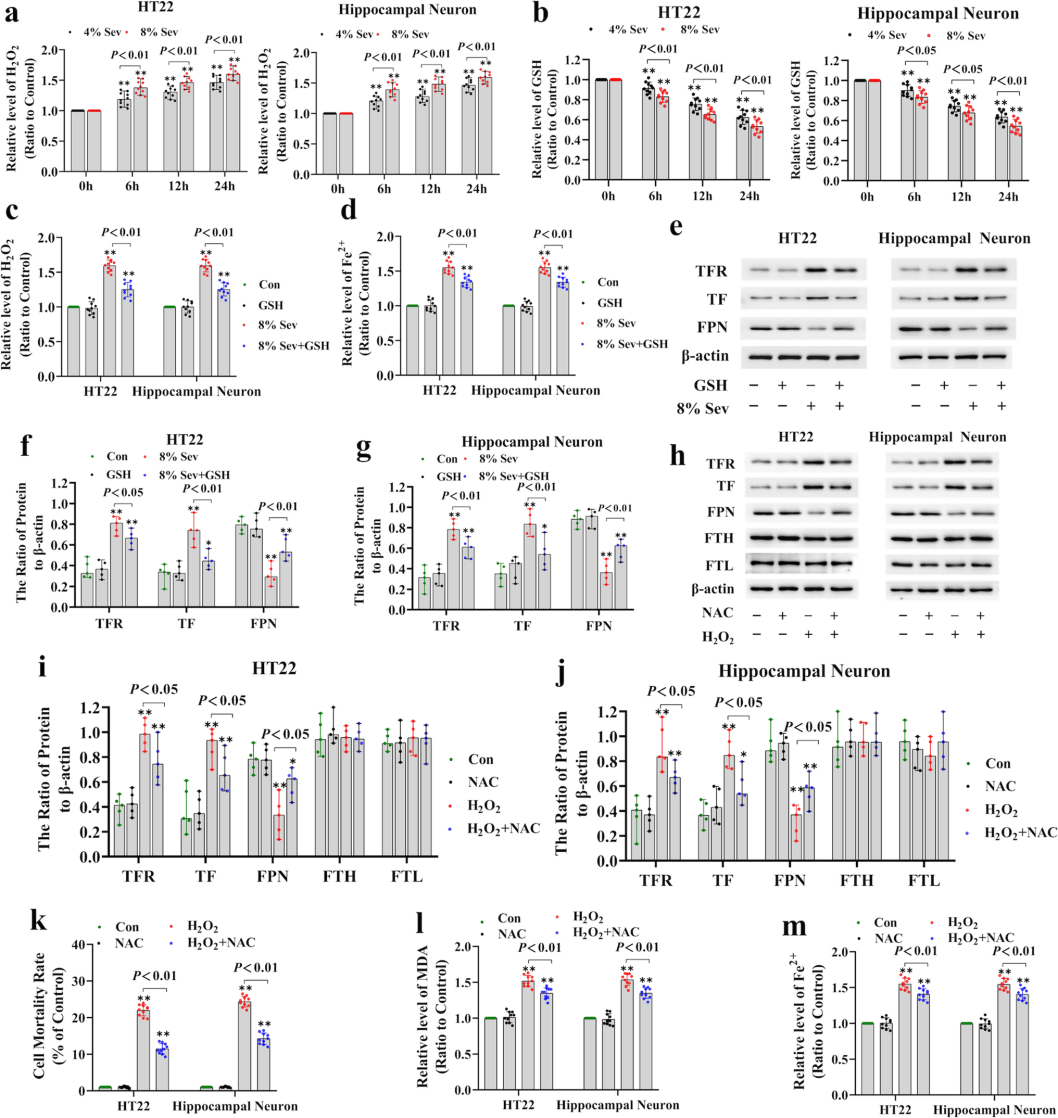
H_2_O_2_ was implicated in sevoflurane-induced neuronal cell ferroptosis via an increase in iron. (**a**) Sevoflurane increased intracellular H_2_O_2_ in a time- and concentration-dependent manner. (**b**) Sevoflurane reduced GSH levels in a time- and concentration-dependent manner. (**c**) Supplementation with GSH (2.5 mM) prevented the increase in H_2_O_2_ caused by 8% sevoflurane exposure for 24 h. (**d**) The iron assay showed that 8% sevoflurane exposure for 24 h increased intracellular iron in neuronal cells, and this effect was suppressed by pretreatment with GSH. (**e–g**) Western blotting revealed that neuronal cells treated with 8% sevoflurane for 24 h had upregulated TFR and TF expression and downregulated FPN expression, and these effects were prevented by GSH. (**h–j**) Western blotting showed that NAC (5 mM) markedly suppressed the upregulation of TFR and TF and downregulation of FPN induced by treatment of neuronal cells with 500 μM H_2_O_2_ for 24 h, but had no significant effect on FTH or FTL. (**k**) The LDH release assay showed that treatment with H_2_O_2_ (500 μM) for 24 h markedly increased the rate of neuronal cell death, which was significantly alleviated by NAC. (**l**) The MDA assay demonstrated that lipid peroxidation caused by exposure of neuronal cells to 500 μM H_2_O_2_ for 24 h was mitigated by the administration of NAC. (**m**) The iron assay showed that neuronal cells treated with H_2_O_2_ at 500 μM for 24 h had increased levels of intracellular ferrous iron, and this effect was inhibited by pretreatment with NAC. Compared with the control group, ^∗^*p* < 0.05, ^∗∗^*p* < 0.01

To determine whether H_2_O_2_ causes the increase in intracellular iron that results in iron-dependent cell death following sevoflurane exposure, an H_2_O_2_ assay was conducted, and the role of H_2_O_2_ in the regulation of the increase in intracellular iron caused by sevoflurane was examined because H_2_O_2_ plays a crucial role in modulating the progression of ferroptosis [22]. We found that sevoflurane increased intracellular H_2_O_2_ in a concentration- and time-dependent manner (Fig. 2a), which was accompanied by a reduction in intracellular GSH (Fig. 2b). In contrast, inhibiting intracellular H_2_O_2_ with GSH (2.5 mM) markedly alleviated the increases in intracellular ferrous iron and lipid peroxidation, in addition to neuronal cell death caused by sevoflurane (Figs. 1d, f and 2c, d). Furthermore, we showed that GSH supplementation reversed the sevoflurane-induced upregulation of TF and TFR and downregulation of FPN (Fig. 2e–g). These data suggest that H_2_O_2_ is an upstream factor in the intracellular iron increase in sevoflurane-induced neuronal ferroptosis. To further confirm the role of H_2_O_2_ in the regulation of ferroptosis, neuronal cells were administered exogenous H_2_O_2_. We found that incubating neuronal cells with exogenous H_2_O_2_ (500 μM) for 24 h significantly promoted cell death, increased intracellular iron and lipid peroxidation, upregulated TFR and TF, and downregulated FPN (Fig. 2h–m). Conversely, we observed that pretreatment with the antioxidant NAC significantly mitigated neuronal cell death and lipid peroxidation caused by H_2_O_2_, resulting in a decrease in intracellular iron, the downregulation of TFR and TF, and the upregulation of FPN (Fig. 2h–m). These results showed that H_2_O_2_ contributed to the sevoflurane-induced increase in intracellular iron by upregulating TFR and TF and downregulating FPN, which led to iron-dependent lipid peroxidation and ferroptosis.

### ER Stress-Mediated ATF3 Activation Promoted Sevoflurane-Induced Neuronal Cell Ferroptosis by Increasing H_2_O_2_

**Fig. 3:**
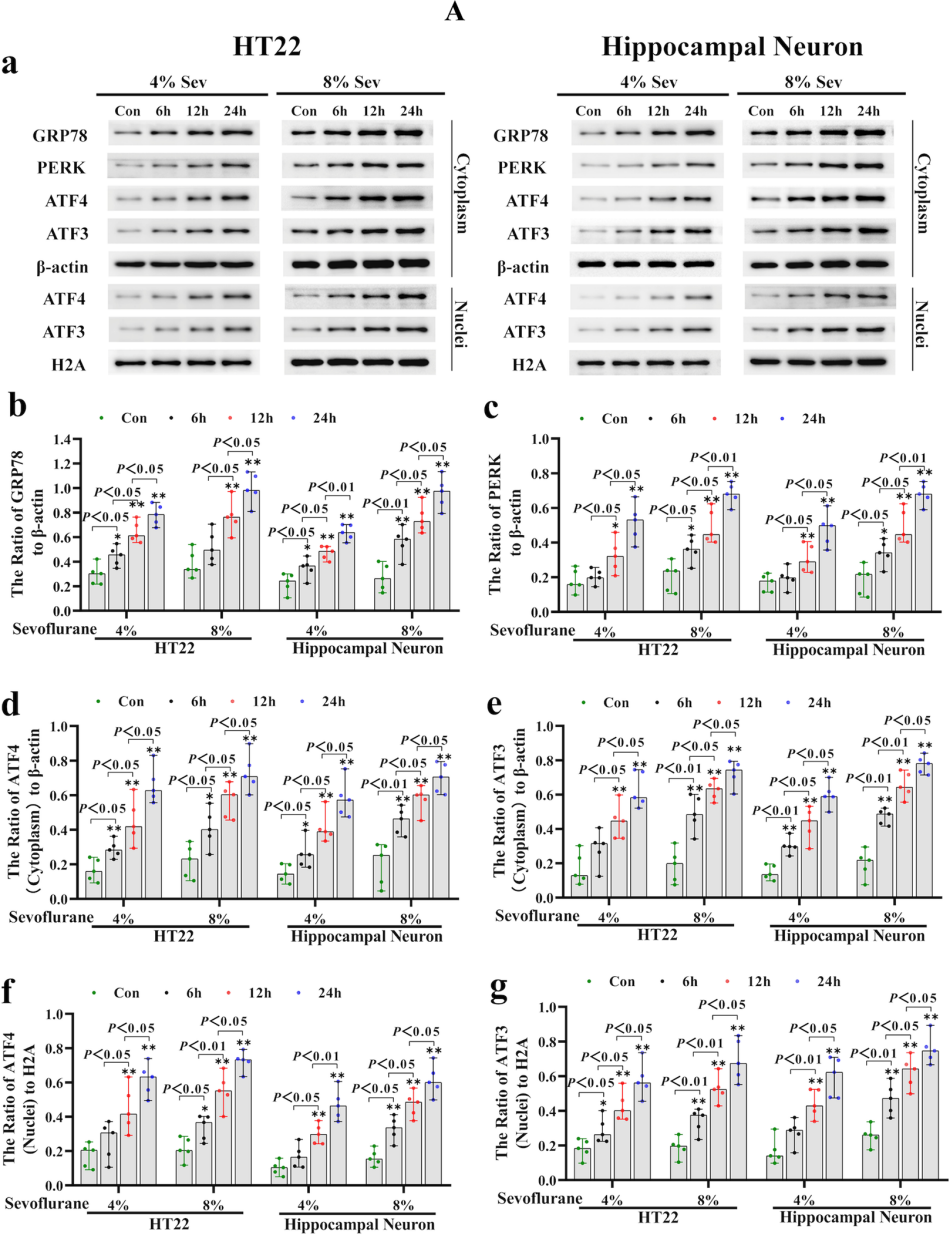

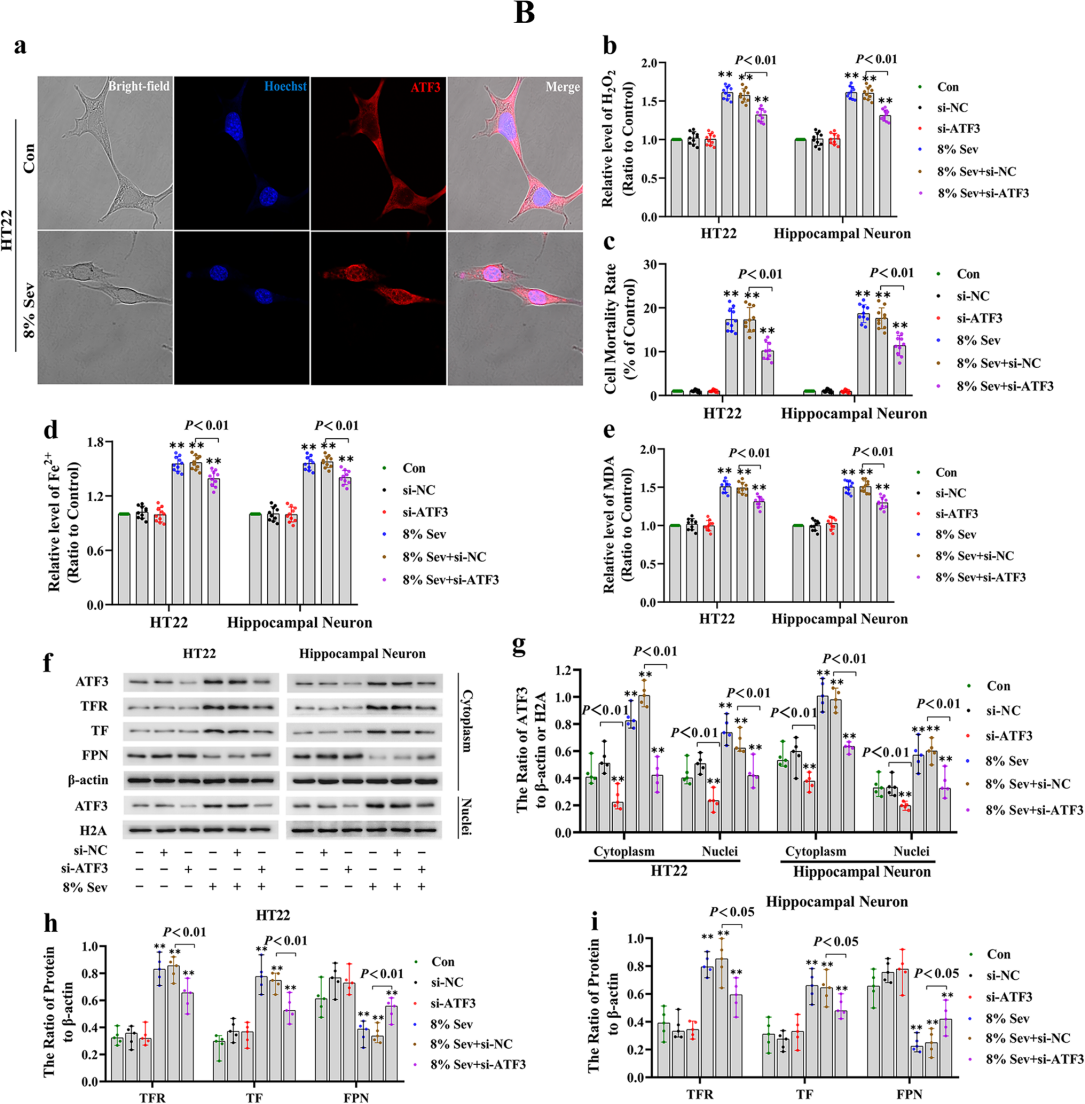
**A** Sevoflurane induced changes in ER stress marker proteins in neuronal cells. (**a**–**g**) Western blotting analysis shown that sevoflurane not only upregulated the levels of ER stress markers proteins GRP78, PERK, ATF4, and ATF3 in neuronal cells in a concentration- and time-dependent manner, but also promoted the nuclear translocation of ATF4 and ATF3. Compared with the control group, ^∗^*p* < 0.05, ^∗∗^*p* < 0.01. (**B**) ATF3 activation promoted sevoflurane-induced neuronal cell ferroptosis by increasing H_2_O_2_. (**a**) Representative confocal microscopy images showed that ATF3 accumulated clearly in the nuclei of HT22 cells following 8% sevoflurane exposure for 24 h in contrast to that in the control group. (**b**) The increase in intracellular H_2_O_2_ in neuronal cells induced by 8% sevoflurane for 24 h was attenuated when ATF3 was knocked down by siRNA. (**c**) The LDH release assay proved that ATF3 silencing suppressed neuronal cell death induced by 8% sevoflurane exposure for 24 h. (**d**) The iron assay demonstrated that ATF3 silencing prevented the increase in ferrous iron in neuronal cells induced by 8% sevoflurane for 24 h. (**e**) MDA assay showed that lipid peroxidation in neuronal cells induced by 8% sevoflurane exposure for 24 h was inhibited by knockdown of ATF3 expression. (**f**–**i**) Western blotting showed that 8% sevoflurane exposure for 24 h not only increased ATF3 expression in the cytoplasm and nucleus of neuronal cells but also upregulated the levels of TFR and TF and downregulated the level of FPN, and these effects were markedly suppressed when neuronal cells were transfected with siRNA ATF3. Compared with the control group, ^∗^*p* < 0.05, ^∗∗^*p* < 0.01

Given that ATF3 can improve intracellular H_2_O_2_ and promote ferroptosis [28, 29, 32], we investigated whether ATF3 activation was involved in sevoflurane-induced neuronal cell ferroptosis by increasing H_2_O_2_. As shown in Fig. 3A (a, e, g), each concentration of sevoflurane and time increased the expression and nuclear translocation of ATF3 in neuronal cells. Consistently, confocal microscopy verified that ATF3 clearly accumulated in the nuclei of neuronal cells following sevoflurane exposure (Fig. 3B (a)). To determine the role of ATF3 in neuronal ferroptosis caused by sevoflurane, siRNA was use to knock down ATF3 in HT22 cells and hippocampal neurons. We observed that knockdown of ATF3 expression suppressed the sevoflurane-induced increase in intracellular H_2_O_2_ and neuronal cell death (Fig. 3B (b, c)). Moreover, the overt increase in intracellular ferrous iron and lipid peroxidation levels, in addition to TF and TFR upregulation and the downregulation of FPN caused by sevoflurane, was mitigated by siRNA-mediated knockdown of ATF3 (Fig. 3B (d–i)). These results suggested that ATF3 activation contributed to sevoflurane-induced neuronal cell ferroptosis by increasing intracellular H_2_O_2_.

**Fig. 4:**
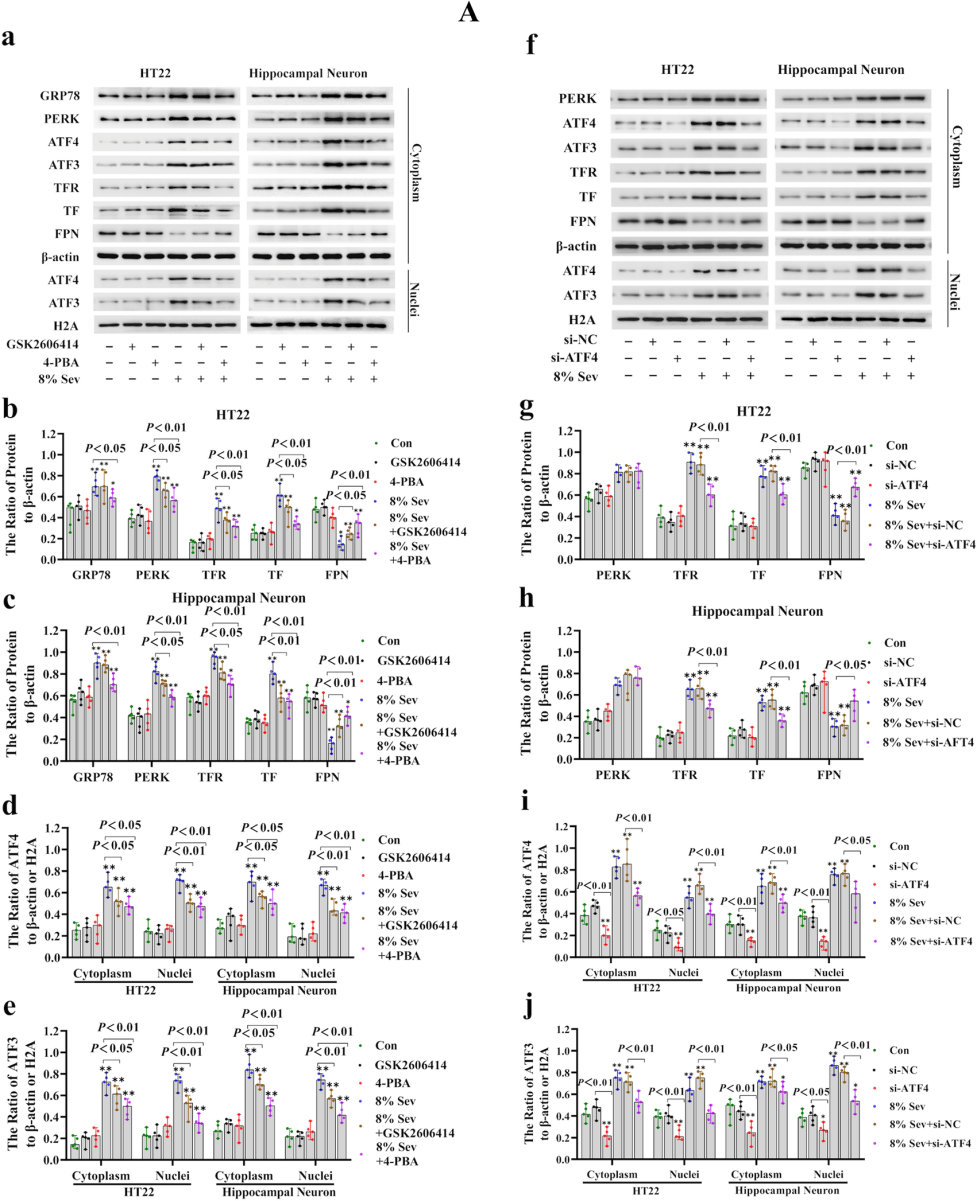

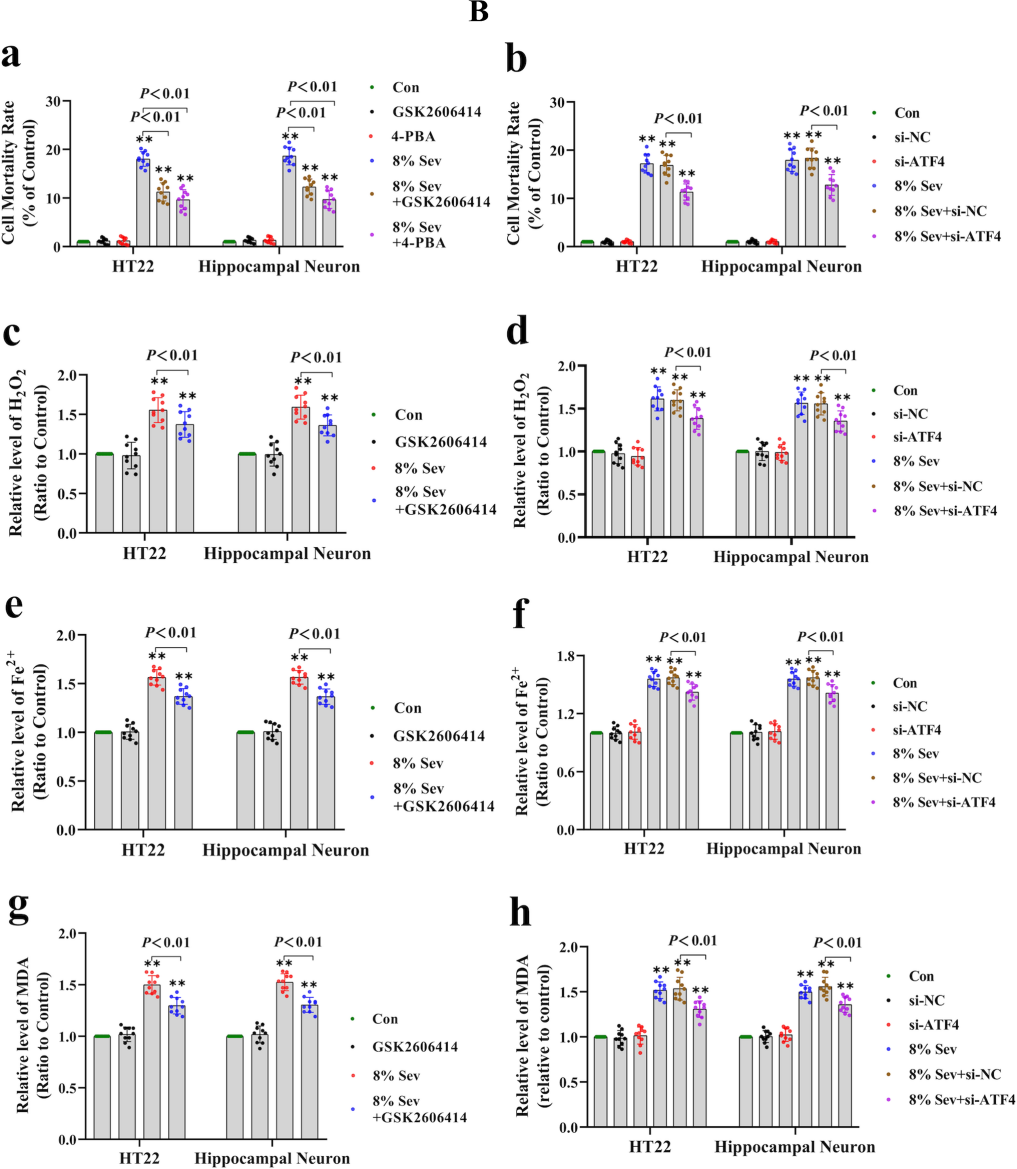
**A** Sevoflurane-induced ATF3 activation was regulated by the ER stress via the PERK/ATF4-mediated pathway. (**a**–**e**) Western blotting analysis revealed that neuronal cells treated with 8% sevoflurane for 24 h markedly induced apparent GRP78, PERK, ATF4, ATF3, TFR, and TF upregulation and FPN downregulation, which were all significantly mitigated by pretreatment with ER stress inhibitor 4-PBA (250 μM) or PERK inhibitor GSK2606414 (1 μM). (**f–j**) Western blotting shown that knockdown of ATF4 prevented the PERK, ATF4, ATF3, TFR, and TF upregulation and FPN downregulation induced by 8% sevoflurane for 24 h. Compared with the control group, ^∗^*p* < 0.05, ^∗∗^*p* < 0.01. (**B**) ER stress-induced PERK/ATF4 pathway participated in sevoflurane-induced neuronal cell ferroptosis. (**a**, **b**) The LDH release assay proved that neuronal cell death induced by 8% sevoflurane exposure for 24 h was alleviated by 4-PBA and GSK2606414 or knockdown of ATF4 expression. (**c**, **d**) The increase in intracellular H_2_O_2_ in neuronal cells induced by 8% sevoflurane for 24 h was reversed by pretreatment with GSK2606414 or siRNA targeting ATF4. (**e**, **f**) The iron assay showed that GSK2606414 pretreatment or ATF4 knockdown suppressed the increase in iron caused by 8% sevoflurane exposure for 24 h. (**g**, **h**) The MDA assay showed that lipid peroxidation in neuronal cells caused by 8% sevoflurane exposure for 24 h was inhibited by GSK2606414 or ATF4 knockdown. Compared with the control group, ^∗^*p* < 0.05, ^∗∗^*p* < 0.01

Considering that ER stress is a major source of H_2_O_2_ and that ATF3 expression can be induced by ER stress through the PERK/ATF4-mediated pathway [28, 29], we examined the role of ER stress in sevoflurane-induced ATF3 activation and neuronal ferroptosis. The results showed that neuronal cells treated with 4% and 8% sevoflurane for 6, 12, and 24 h exhibited increases in proteins representing ER stress markers, including GRP78, PERK, and ATF4, and the effect was dependent on time and concentration (Fig. 3A (a–d, f)); these effects were abrogated by the ER stress inhibitor 4-PBA or the PERK inhibitor GSK2606414 (Fig. 4A (a–d)). Moreover, we observed that neuronal death caused by sevoflurane was ameliorated when the cells were pretreated with 4-PBA and GSK2606414 or siRNA targeting ATF4 (Fig. 4B (a, b)). These results suggested that the ER stress-related PERK/ATF4 pathway participated in sevoflurane-induced neuronal cell death. Furthermore, the sevoflurane-induced upregulation of ATF3 in the cytoplasm and nucleus was markedly suppressed by 4-PBA, GSK2606414, or knockdown of ATF4 expression (Fig. 4A (a, e, f, j)), indicating that sevoflurane-induced ATF3 activation was regulated by the PERK/ATF4 pathway in response to ER stress. In addition, we found that the sevoflurane-induced increases in intracellular H_2_O_2_, iron, and lipid peroxidation, as well as the upregulation of TFR and TF and downregulation of FPN, were significantly alleviated by GSK2606414 or ATF4 knockdown (Fig. 4A (a–c, f–h) and 4B (c–h)). Collectively, these findings revealed that ER stress and PREK/ATF4-mediated ATF3 activation promoted sevoflurane-induced neuronal cell ferroptosis by increasing intracellular H_2_O_2_.

### ATF3 Promoted the Sevoflurane-Induced Increase in H_2_O_2_ by Activating NOX4 and Suppressing Catalase, GPX4, and SLC7A11

**Fig. 5:**
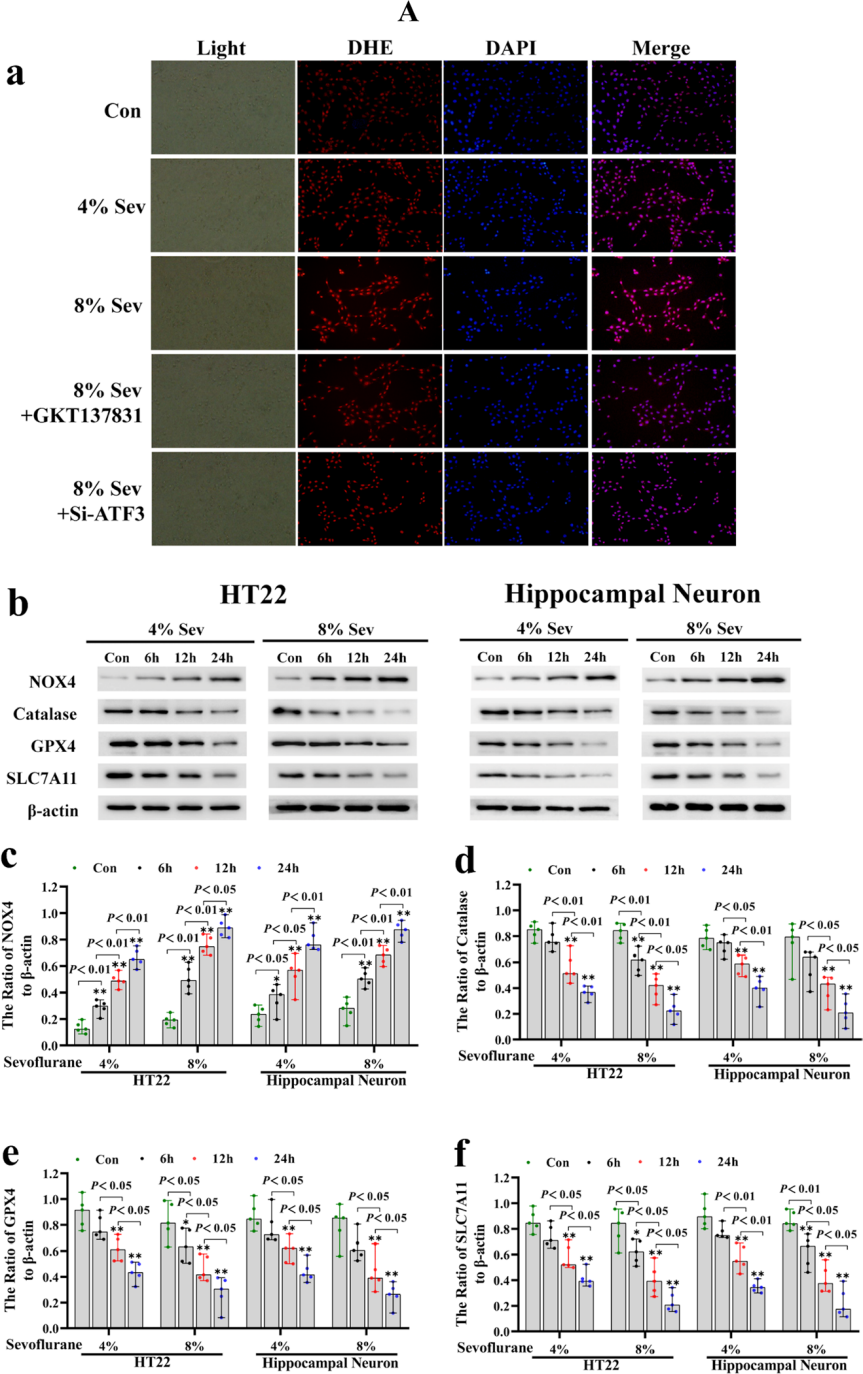

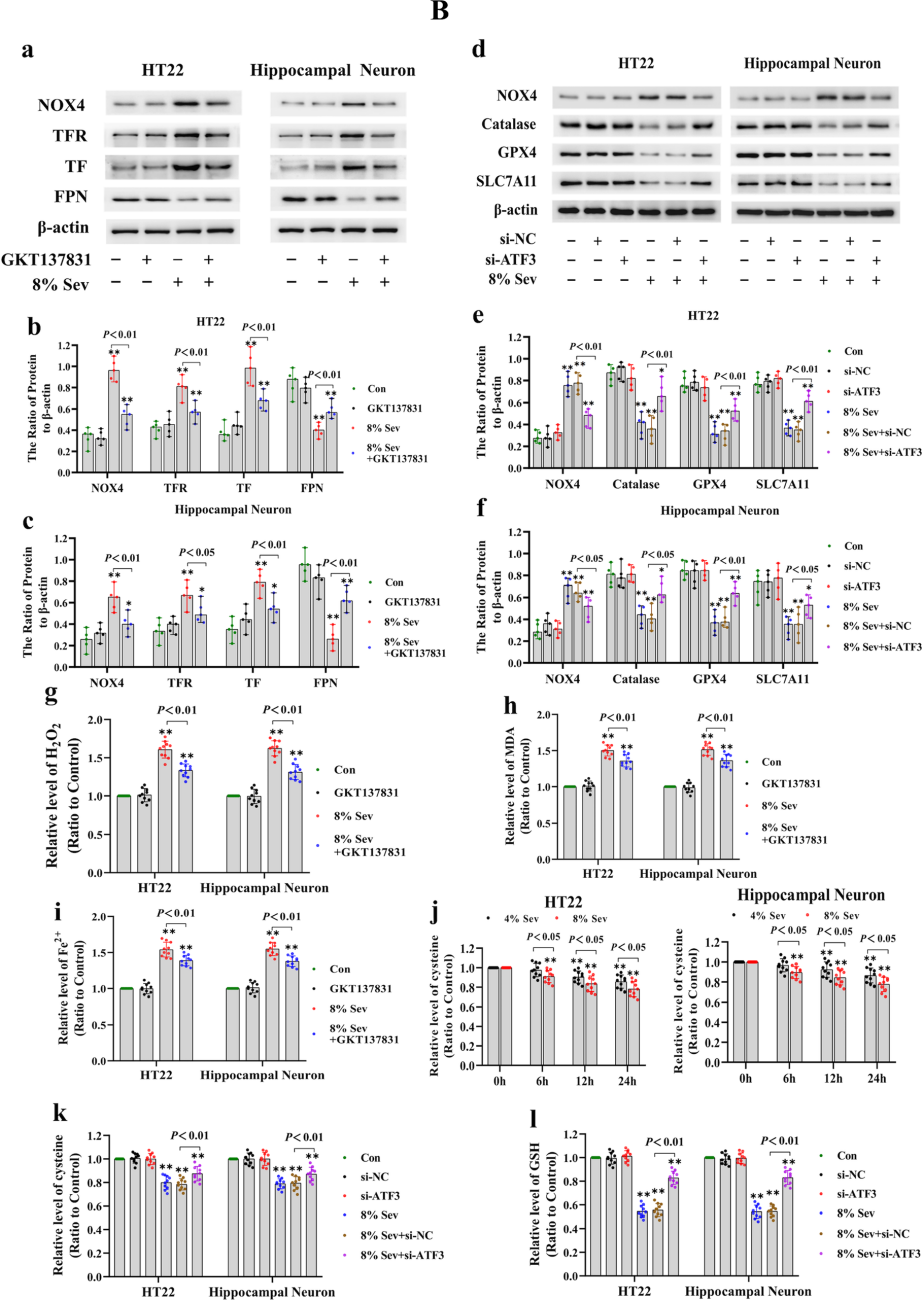
**A** Sevoflrane induced the increase in superoxide and upregulation of NOX4 and downregulation of calatase, GPX4, and SLC7A11 in neuronal cells. (**a**) Fluorescence microscopy revealed that the levels of superoxide, as shown by the intensity of dihydroethidium (DHE) fluorescence, were significantly increased in neuronal cells following 4% sevoflurane exposure for 24 h and were further elevated when neuronal cells were subjected to 8% sevoflurane for 24 h, and these effects were counteracted by the NOX4 inhibitor GKT137831 (50 μM) or knockdown of ATF3 expression. (**b**–**f**) Western blotting demonstrated that sevoflurane upregulated NOX4 and downregulated catalase, GPX4, and SLC7A11 in a time- and concentration-dependent manner. Compared with the control group, ^∗^*p* < 0.05, ^∗∗^*p* < 0.01. (**B**) ATF3 promoted the sevoflurane-induced increase in H_2_O_2_ by activating NOX4 and suppressing catalase, GPX4, and SLC7A11. (**a**–**c**) Western blotting revealed that neuronal cells treated with 8% sevoflurane for 24 h had upregulated NOX4, TFR, and TF expression and downregulated FPN expression, and these effects were prevented by GKT137831. (**d**–**f**) Western blotting showed that ATF3 silencing markedly suppressed the upregulation of NOX4 and downregulation of catalase, GPX4, and SLC7A11 in neuronal cells caused by 8% sevoflurane for 24 h. (**g**) Supplementation with GKT137831 prevented the increase in H_2_O_2_ caused by 8% sevoflurane exposure for 24 h. (**h**) MDA assay showed that lipid peroxidation induced by 8% sevoflurane for 24 h in neuronal cells was mitigated in the existence GKT137831. (**i**) The iron assay showed that 8% sevoflurane exposure for 24 h increased intracellular iron in neuronal cells, and this effect was suppressed by pretreatment GKT137831. (**j**) Sevoflurane induced a concentration- and time-dependent decrease in cysteine in neuronal cells. (**k**, **l**) The depletion of intracellular cysteine and GSH induced by 8% sevoflurane for 24 h was markedly suppressed when neuronal cells were transfected with siRNA ATF3. Compared with the control group, ^∗^*p* < 0.05, ^∗∗^*p* < 0.01

To examine why ATF3 could promote the sevoflurane-induced increase in intracellular H_2_O_2_, we investigated the role of ATF3 in regulating NADPH oxidase 4 (NOX4) because NOX4 is mainly located in the endoplasmic reticulum and can generate superoxide, which is subsequently transformed into H_2_O_2_ during ER stress [42]. The level of sevoflurane-induced intracellular superoxide was detected by using dihydroethidium (DHE). Fluorescence microscopy revealed that the levels of superoxide, as indicated by the intensity of DHE fluorescence, were significantly increased in neuronal cells after 4% sevoflurane exposure for 24 h and were further elevated when neuronal cells were subjected to 8% sevoflurane (Fig. 5A (a)). Consistently, our results showed that sevoflurane exposure was linked with the upregulation of NOX4 in neuronal cells (Fig. 5A (b, c)). In contrast, pretreatment with the NOX4 inhibitor GKT137831 markedly counteracted the sevoflurane-induced upregulation of NOX4 and increase in superoxide and H_2_O_2_ in neuronal cells (Fig. 5A (a) and 5B (a–c, g)), suggesting that NOX4 promoted the sevoflurane-induced increase in intracellular H_2_O_2_. Moreover, the increases in iron and lipid peroxidation, as well as the upregulation of TFR and TF and downregulation of FPN induced by sevoflurane, were markedly alleviated by GKT137831 (Fig. 5B (a–c, h, i)). This further confirmed that H_2_O_2_ contributed to sevoflurane-induced iron-dependent neuronal cell death. Considering that catalase is a known intracellular H_2_O_2_ scavenging enzyme [43], we then verified the role of catalase in the increase in intracellular H_2_O_2_ induced by sevoflurane. We observed that catalase levels were significantly decreased by sevoflurane (Fig. 5A (b, d)), suggesting that catalase was involved in the sevoflurane-induced increase in H_2_O_2_. Notably, we found that ATF3 silencing markedly prevented the sevoflurane-induced increase in NOX4 and decrease in catalase, as well as the generation of superoxide and H_2_O_2_ (Fig. 5A (a) and 5B (d–f)). Thus, our findings indicated that ATF3 promoted the sevoflurane-induced increase in H_2_O_2_ by upregulating NOX4 and downregulating catalase.

**Fig. 6:**
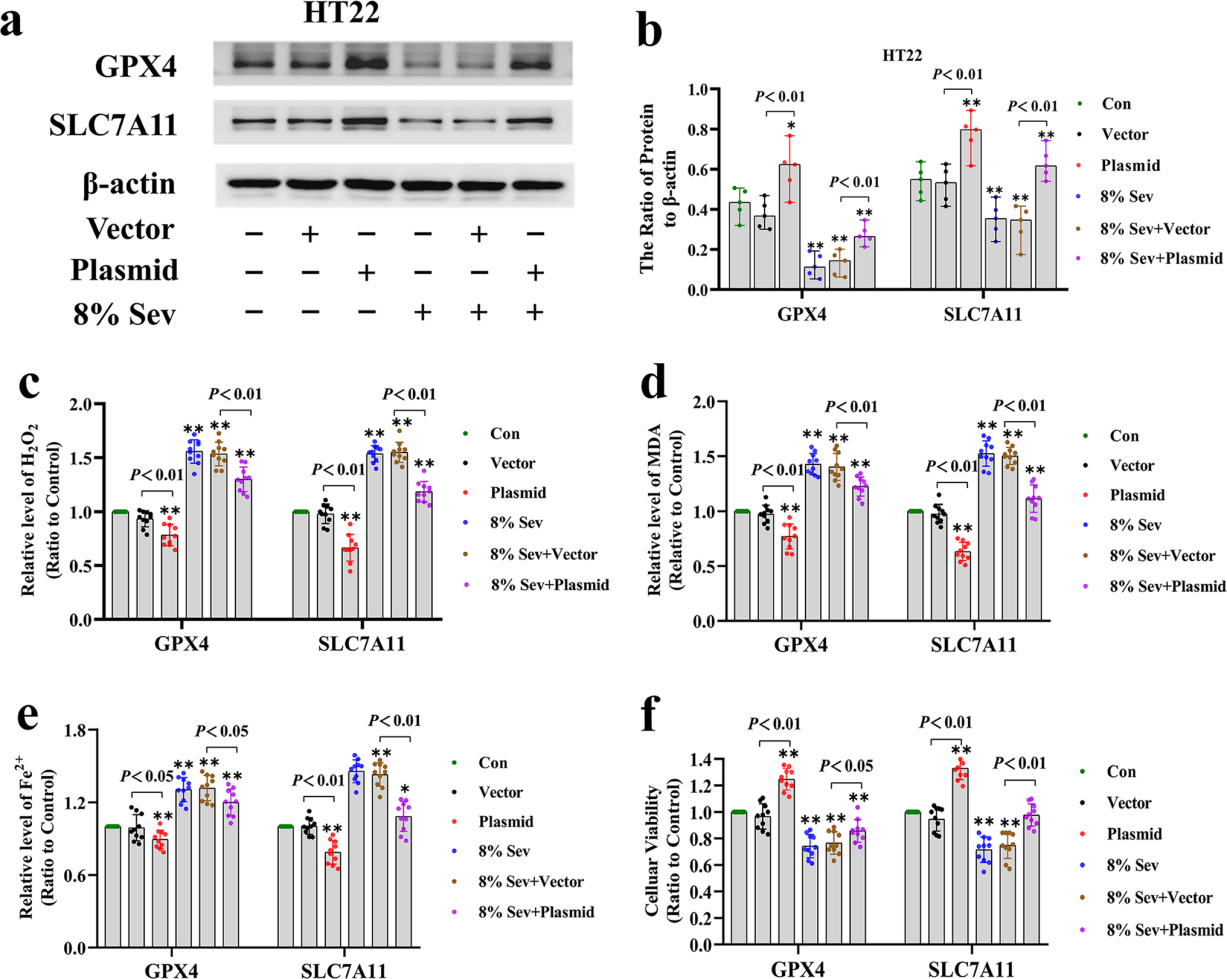
Overexpression of GPX4 and SLC7A11 alleviated sevoflurane-induced ferroptosis in HT22 cells. (**a**, **b**) The representative Western blot bands and semiquantitative analysis of GPX4 and SLC7A11 in each group. (**c**–**f**) Overexpression of GPX4 and SLC7A11 not only significantly suppressed the increase in intracellular H_2_O_2_ and lipid peroxidation induced by 8% sevoflurane for 24 h but also markedly attenuated the increase in ferrous iron and the decrease in cellular viability after sevoflurane exposure.  Compared with the control group, ∗*p*＜0.05, ∗∗*p*＜0.01

Intracellular H_2_O_2_ can be scavenged by glutathione peroxidase 4 (GPX4) through the consumption of GSH, which is synthesized mainly from cysteine that can be converted from cystine [32, 44]. To examine the role of GPX4 in H_2_O_2_ accumulation induced by sevoflurane, we examined sevoflurane-induced alterations in GSH and GPX4. The results showed that sevoflurane decreased GPX4 in neuronal cells, and there was a decrease in GSH and an increase in intracellular H_2_O_2_ (Figs. 2b, c and 5A (b, e)). Then, we examined the expression of SLC7A11, which is a unique subunit of amino acid antiporter system Xc − that facilitates the exchange between internal glutamate and extracellular cysteine [32, 45]. Our results showed that sevoflurane exposure decreased SLC7A11 and cysteine levels in neuronal cells in a time- and concentration-dependent manner (Fig. 5A (b, f) and 5B (j)). Moreover, overexpression of GPX4 and SLC7A11 not only significantly suppressed the increase in intracellular H_2_O_2_ and lipid peroxidation induced by sevoflurane but also markedly attenuated the increase in ferrous iron and the decrease in cellular viability after sevoflurane exposure (Fig. 6a–f). These results further confirmed that the inhibition of GPX4 and SLC7A1 in iron-dependent neuronal cell death processes is the main mechanism by which sevoflurane induces an increase in intracellular H_2_O_2_. Notably, the sevoflurane-induced downregulation of GPX4 and SLC7A11, as well as the depletion of cysteine and GSH, was markedly alleviated when neuronal cells were transfected with siRNA against ATF3 (Fig. 5B (d–f, k, l)). These data demonstrated that ATF3 promoted the sevoflurane-induced increase in H_2_O_2_ by suppressing GPX4 and SLC7A11.

### ER Stress-Mediated ATF3 Activation Contributed to Sevoflurane-Induced Neuronal Ferroptosis in the Hippocampus of Neonatal Mice

**Fig. 7:**
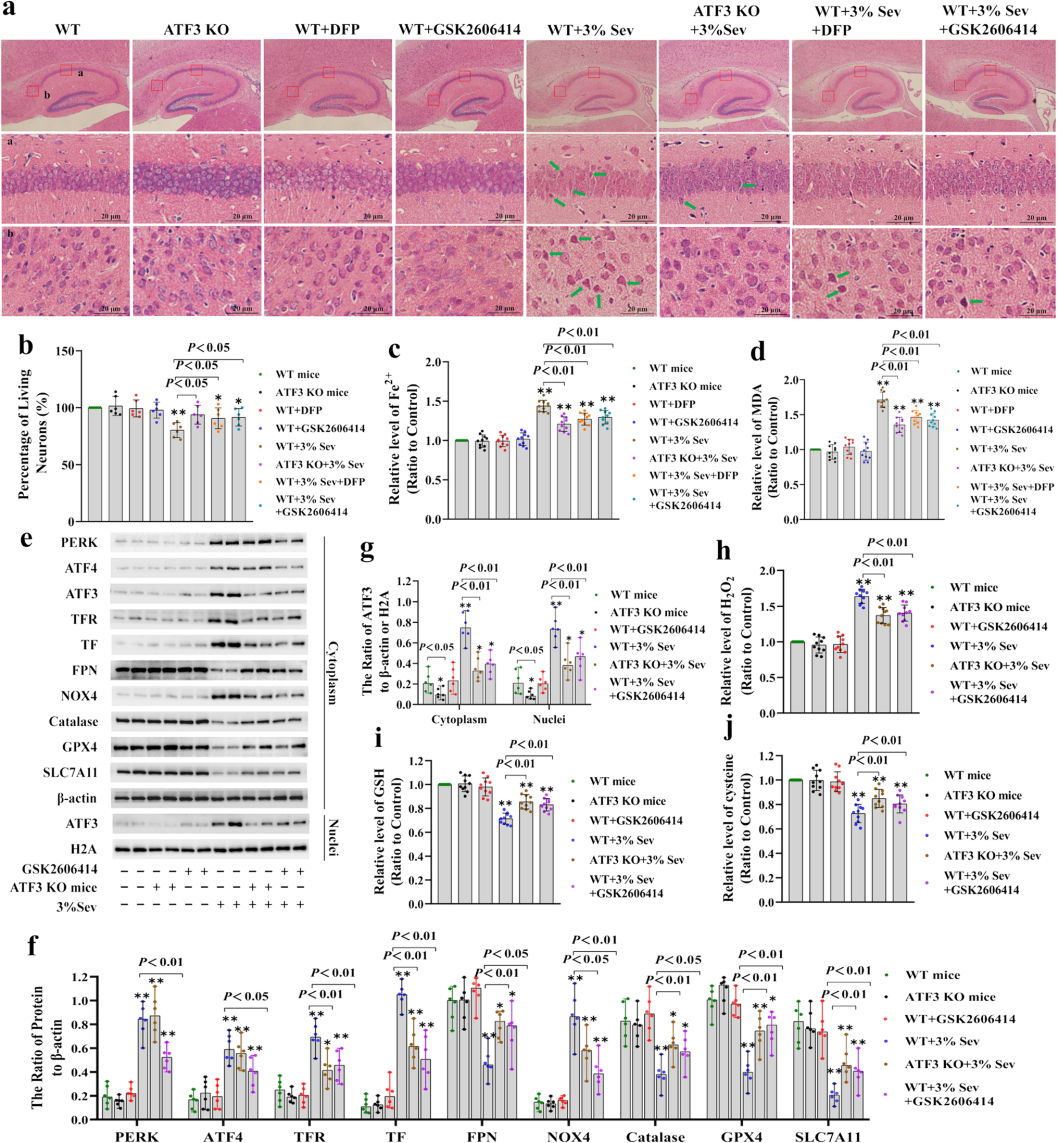
ATF3 contributed to sevoflurane-induced neuronal ferroptosis in the hippocampus of neonatal mice. (**a**) Representative images of hippocampal neurons stained with HE on day 7 after anesthesia, and the mice had received 3% sevoflurane exposure for 2 h on postnatal day (P) 6, P7, and P8. Scale bar = 20 µm. Compared with that in the control group, sevoflurane caused pyramidal neuron death (green arrow) in the hippocampal CA1 region, which was characterized by sparse and disordered neuronal structures, cell shrinkage, morphologically pink cytoplasm, and pyknotic nuclei, and these effects were ameliorated by GSK2606414 (50 mg/kg) and DFP (75 mg/kg) or ATF3 silencing. (**b**) Statistical analysis revealed that the percentage of living pyramidal neurons in the hippocampal CA1 region was 80% on day 7 after sevoflurane exposure, and pretreatment with GSK2606414 and DFP or ATF3 silencing markedly rescued the sevoflurane-induced decrease in living neurons. (**c**, **d**) Repeated sevoflurane exposure significantly increased intracellular iron and MDA levels in the hippocampi of neonatal mice, and these effects were prevented by GSK2606414 and DFP or ATF3 silencing. (**e**–**g**) Western blotting showed that sevoflurane exposure upregulated ATF3, TFR, TF, and NOX4 and downregulated FPN, catalase, GPX4, and SLC7A11, and these effects were reversed by the PERK inhibitor GSK2606414 or ATF3 silencing. (**h**) GSK2606414 pretreatment or ATF3 silencing significantly prevented the sevoflurane-induced accumulation of H_2_O_2_ in the hippocampi of neonatal mice. (**i**, **j**) Repeated sevoflurane exposure caused the depletion of GSH and cysteine in the hippocampi of neonatal mice, and this effect was mitigated by pretreatment with GSK2606414 or ATF3 silencing. Compared with the control group, ^∗^*p* < 0.05, ^∗∗^*p* < 0.01

Previous studies have shown that exposing neonatal mice to 3% sevoflurane for 2 h every day for three consecutive days could result in neuronal cell death and persistent cognitive impairment [36, 37]. Thus, neonatal mice were treated with 3% sevoflurane for 2 h per day on postnatal days (P) 6, P7, and P8 to determine whether ferroptosis was involved in sevoflurane-induced neonatal neuronal death. Consistent with previous reports [46], the O_2_ partial pressure, pH, and CO_2_ partial pressure in neonatal mice exposed to sevoflurane for 2 h were not significantly different from those in the control group (Table 1). Pathological examination of the hippocampus on day 7 after sevoflurane anesthesia by HE staining showed that sevoflurane exposure markedly increased hippocampal neuronal death or injury, which was characterized by sparsely and morphologically pink cytoplasm, disordered neuronal structure, cell shrinkage, and pyknotic nuclei (Fig. 7a). Statistical analysis revealed that on day 7 after sevoflurane exposure, 80% of pyramidal neurons in the hippocampal CA1 region were alive (Fig. 7b). These results suggested that repeated sevoflurane exposure induced visible hippocampal neuronal death in neonatal mice. Moreover, we observed that repeated sevoflurane exposure increased ferrous iron and MDA levels in the hippocampi of neonatal mice (P8) at 6 h, and these effects were diminished by pretreatment with the iron chelator deferiprone (DFP) (Fig. 7c, d). Furthermore, DFP pretreatment markedly attenuated sevoflurane-induced morphological changes and significantly improved the number of pyramidal neurons that were still alive in the hippocampal CA1 region (Fig. 7a, b). These results showed that repeated sevoflurane exposure induced hippocampal neuronal cell ferroptosis in the developing brain.

**Table 1:** Arterial blood gas after 2 h of 3% sevoflurane or no anesthesia in P8 mice pups (*n* = 6 per group)

	WT mice	ATF3 KO mice	WT + DFP	WT + GSK2606414	WT + 3% Sev	ATF3 KO + 3% Sev	WT + 3% Sev + DFP	WT + 3% Sev + GSK2606414
pH	7.399 ± 0.017	7.392 ± 0.014	7.395 ± 0.014	7.395 ± 0.013	7.397 ± 0.016	7.389 ± 0.020	7.390 ± 0.018	7.388 ± 0.016
PaCO_2_ (mmHg)	29.33 ± 1.211	30.00 ± 1.673	29.50 ± 1.871	29.83 ± 1.941	30.33 ± 2.066	30.00 ± 2.098	30.33 ± 2.251	29.17 ± 2.317
PaO_2_ (mmHg)	97.00 ± 5.060	96.83 ± 6.853	99.50 ± 5.958	95.00 ± 4.858	96.67 ± 6.154	98.5 ± 5.244	97.17 ± 5.947	100.50 ± 5.125

Data are presented as mean ± SD

*PaCO*_*2*_, partial pressure of arterial carbon dioxide; *PaO*_*2*_, partial pressure of arterial oxygen

To verify whether ER stress-mediated ATF3 activation was involved in sevoflurane-induced neonatal hippocampal neuronal ferroptosis, wild-type (WT) mice treated with GSK2606414 or ATF3-knockout (KO) mice were exposed to 3% sevoflurane for 2 h daily from P6 to P8. We observed that repeated sevoflurane exposure increased PERK, ATF4, and ATF3 levels in the hippocampi of neonatal mice (Fig. 7e–g), indicating that sevoflurane promoted ER stress and PERK/ATF4/ATF3 pathway activation in the hippocampi of neonatal mice. Moreover, pretreatment with GSK2606414 or ATF3 silencing not only significantly reduced sevoflurane-induced neuronal cell death and the increase in ferrous iron and MDA levels but also suppressed the upregulation of TF and TFR and downregulation of FPN in the hippocampi of neonatal mice (Fig. 7a–g). These results suggested that ER stress-mediated ATF3 activation participated in sevoflurane-induced hippocampal neuronal ferroptosis in the developing brain. Furthermore, we found that the sevoflurane-induced increase in H_2_O_2_, upregulation of NOX4 and downregulation of SLC7A11, GPX4, and catalase, as well as the reductions in GSH and cysteine in the hippocampi of neonatal mice were all significantly mitigated by GSK2606414 treatment or ATF3 silencing (Fig. 7e–j). These results further confirmed that ER stress-mediated ATF3 activation contributed to sevoflurane-induced hippocampal neuronal ferroptosis via H_2_O_2_ accumulation and resultant iron overload in neonatal mice.

### ATF3-Mediated Ferroptosis Participated in Sevoflurane-Induced Spatial Memory Impairment in Neonatal Mice

**Fig. 8:**
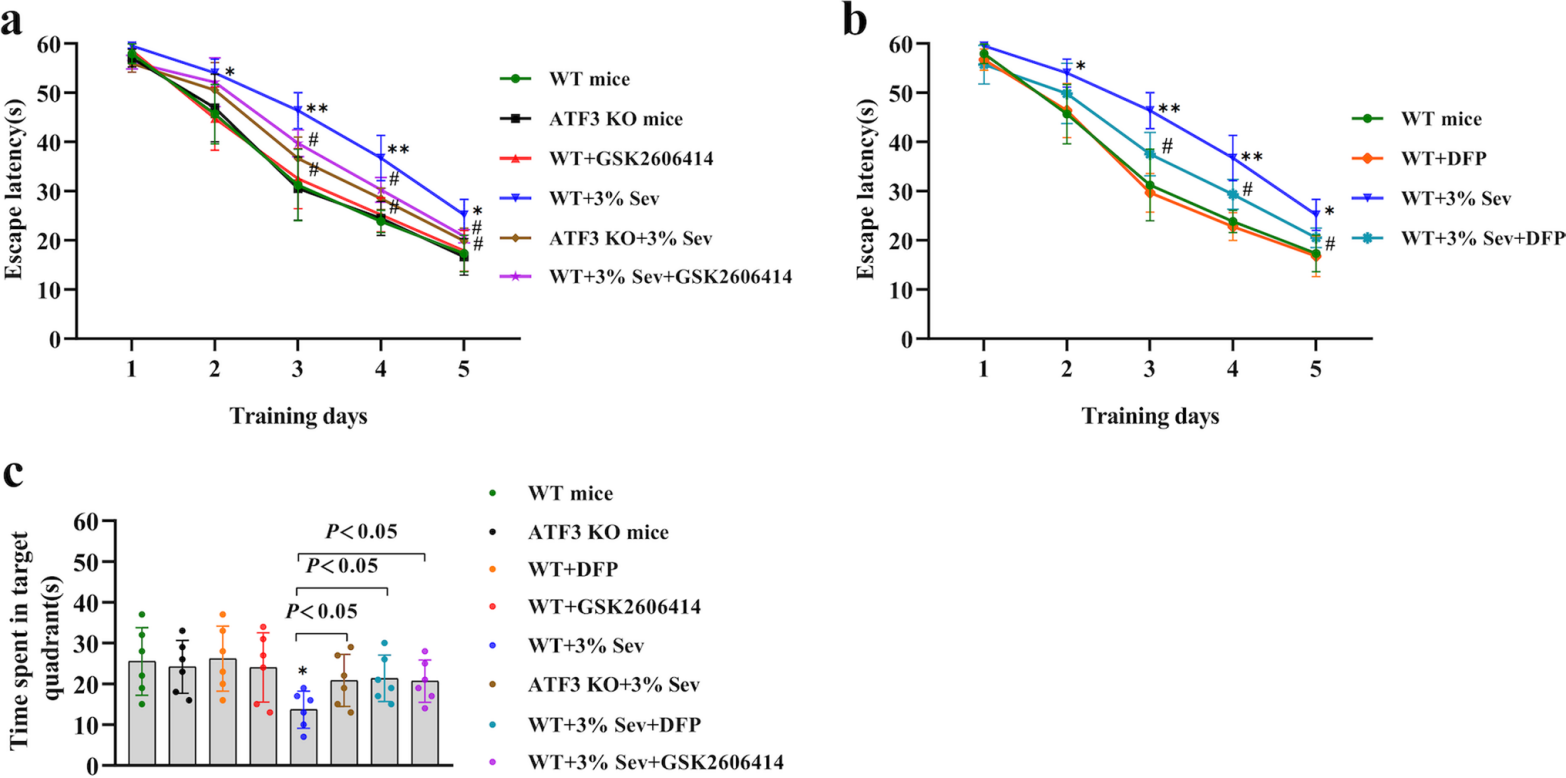
ER stress and ATF3 activation-mediated ferroptosis participated in sevoflurane-induced spatial memory impairment in neonatal mice. (**a**, **b**) Escape latency was longer in the sevoflurane group in the spatial training tests on days 3, 4, and 5 than in the control group, but mice treated with GSK2606414 and DFP or ATF3 silencing had shorter latencies than those in the sevoflurane group. (**c**) Statistical analysis revealed that mice exposed to sevoflurane spent markedly less time in the target quadrant than mice in the control group during the probe trial on day 6, whereas mice treated with GSK2606414 and DFP or ATF3 silencing spent more time in the target quadrant. Compared with the control group, ^∗^*p* < 0.05, ^∗∗^*p* < 0.01; compared with the sevoflurane group, ^#^*p* < 0.05, ^##^*p* < 0.01. Data are represented as the mean ± SD (*n* = 6 mice per group)

Given the important role of the hippocampus in spatial learning and memory, we used the Morris water maze test to evaluate spatial learning and memory performance on postnatal day 31 in mice that had received repeated sevoflurane exposures on the P6, P7, and P8. As training proceeded, the mice had visibly reduced latencies to detect the hidden platform, suggesting that the mice were learning from daily practice. The typical swimming tracks suggested that mice exposed to sevoflurane had markedly prolonged escape latencies in the spatial training tests on days 3, 4, and 5 and significantly reduced times in the indicated quadrant in the probing experiment on day 6 compared with those in the control group (Fig. 8a–c). In contrast, the mice treated with GSK2606414 and DFP or ATF3 silencing searched for the hidden platform in a more appropriate way, resulting in a shorter delay in the time to find the hiding platform and more time in the target quadrant in contrast to mice in the sevoflurane group (Fig. 8a–c). Therefore, our findings suggested that ER stress and ATF3 activation-mediated ferroptosis accounted for the spatial memory impairment caused by repeated sevoflurane exposure in neonatal mice.

## Discussion

**Fig. 9:**
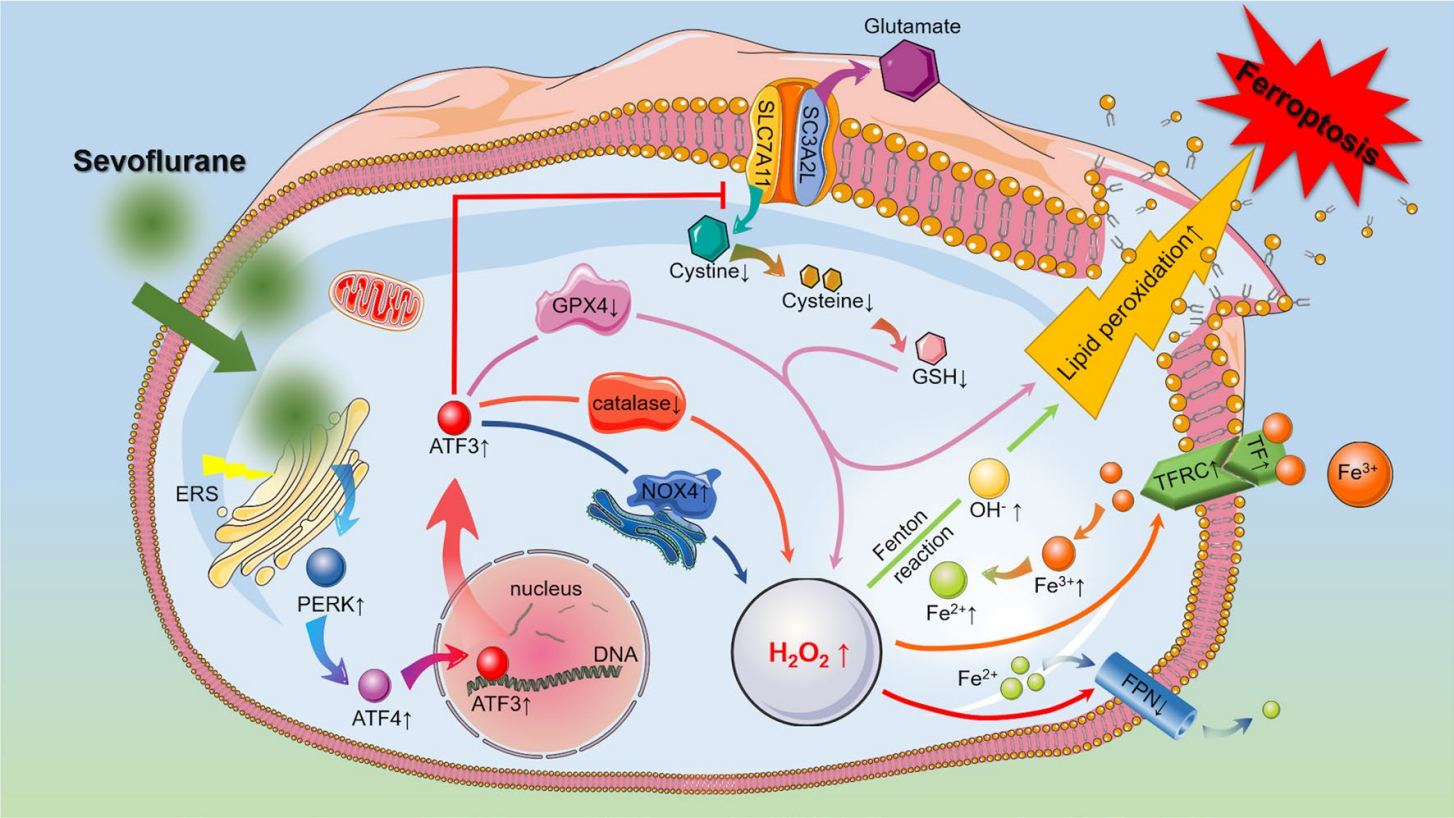
Schematic diagram of the role of ATF3 in sevoflurane-induced neuronal cell ferroptosis. In the developing brain, increases in iron and H_2_O_2_ caused by sevoflurane exposure were linked to the upregulation and nuclear translocation of ATF3 in response to ER stress activation. ATF3 promoted sevoflurane-induced H_2_O_2_ by activating NOX4 and inhibiting catalase, GPX4, and SLC7A11. The accumulation of H_2_O_2_ not only improved intracellular iron by upregulating TFR and TF and downregulating FPN but also reacted with ferrous iron through the Fenton reaction to produce hydroxyl radicals and lipid peroxidation, which accounted for neuronal cell death. Therefore, ER stress-mediated ATF3 activation contributes to the sevoflurane-induced accumulation of H_2_O_2_ and iron overload, leading to iron-dependent cell death (ferroptosis)

In neonates and infants aged 1–6 months, the minimal alveolar concentrations (MACs) of sevoflurane are 3.3% and 3.2%, respectively [47]. In clinical practice, sevoflurane is commonly used in pediatric anesthesia at concentrations ranging from 0.5 to 2.5 MAC, and accordingly, we used 3% (0.9 MAC) sevoflurane in vivo study and 4% (1.2 MAC) or 8% (2.4 MAC) sevoflurane in vitro study. In the present investigation, we observed that sevoflurane induced death in neuronal cells and the hippocampi of neonatal mice. Neuronal cell death occurred as a result of iron-dependent lipid peroxidation secondary to ferrous iron and H_2_O_2_ accumulation, which was consistent with the characteristics of ferroptosis. Furthermore, we observed that the increases in iron and H_2_O_2_ caused by sevoflurane exposure were associated with the upregulation and nuclear translocation of ATF3 in response to ER stress and PERK/ATF4 pathway activation. Knockdown of ATF3 alleviated iron-dependent lipid peroxidation, which inhibited sevoflurane-induced neonatal neuronal ferroptosis in vitro and in vivo. Mechanistically, ATF3 promoted sevoflurane-induced H_2_O_2_ accumulation by activating NOX4 and inhibiting catalase, GPX4, and SLC7A11 expression. Additionally, the increase in H_2_O_2_ was accompanied by the upregulation of TFR and downregulation of FPN, which linked iron overload to ferroptosis induced by sevoflurane. Therefore, our results demonstrated that ER stress-mediated ATF3 activation contributed to sevoflurane-induced neonatal neuronal ferroptosis via H_2_O_2_ and resultant iron overload (Fig. 9).

Ferroptosis is a form of programmed cell death that is characterized by an increase in intracellular ferrous iron and resultant lipid peroxidation, which peroxidizes PUFAs on the cell membrane [12]. In the present investigation, we observed that sevoflurane exposure induced death in neuronal cells and the hippocampi of neonatal mice, which was accompanied by increases in intracellular iron and the lipid peroxidation product MDA. The sevoflurane-induced increase in MDA was suppressed when the excess iron was scavenged by the iron chelator DFO, whereas inhibiting MDA levels with the lipophilic antioxidants Fer-1 or Lip-1 markedly reversed sevoflurane-induced neuronal cell death. Moreover, transmission electronic microscopy revealed that the morphological alterations in neuronal cell membranes and organelles caused by sevoflurane were consistent with the features of ferroptosis [48], distinguishing it from apoptosis, necroptosis, and autophagy. Therefore, our data showed that ferroptosis mediated neuronal cell death caused by sevoflurane in the developing brain.

Intracellular iron overload is considered an essential prerequisite for ferroptosis [12]. Cells maintain appropriate intracellular iron levels through iron regulatory proteins that mediate iron uptake, storage, and export [41]. Transferrin receptor (TFR) can take up extracellular ferric iron into intracellular destinations through the iron-TF-TFR complex and ferritin (FT), which is composed of a light chain (FTL) and heavy chain (FTH), stores excess iron. As an iron efflux pump, ferroportin (FPN) is responsible for exporting iron, and after being activated, FPN subsequently decreases intracellular iron [41]. In contrast to previous reports [11] showing that sevoflurane suppressed the expression of TFR in developing brains, we found that sevoflurane exposure was linked to the upregulation of TFR and TF and the downregulation of FPN in neuronal cells and neonatal mouse hippocampi. Although sevoflurane has been reported to upregulate ferritin in neonatal neuronal cells [11], no significant changes in FTL or FTH were found in this study. Thus, sevoflurane improved intracellular iron in developing brains by upregulating TFR and TF and downregulating FPN.

Intracellular H_2_O_2_ plays a crucial role in the progression of ferroptosis. H_2_O_2_ can react with ferrous iron through the Fenton reaction to generate hydroxyl radicals, which have high peroxidative toxicity to PUFA and upregulate TFR, which leads to an increase in intracellular iron [22, 23]. In comparison to the mature brain, the immature brain contains higher concentrations of PUFA and iron and lower levels of antioxidants, making it particularly sensitive to the consequences of iron-dependent lipid peroxidation [13, 14, 19, 20]. Neuronal death following sevoflurane exposure is thought to be partly mediated by the accumulation of excess intracellular reactive oxygen species, which results in oxidative stress and lipid peroxidation [49, 50]. Our findings showed that in vitro and in vivo exposure to sevoflurane induced neonatal neuronal cell death, which was linked to increases in intracellular H_2_O_2_, iron, and lipid peroxidation. In contrast, inhibiting H_2_O_2_ with the antioxidant GSH markedly suppressed the increases in iron and lipid peroxidation, as well as neuronal cell death induced by sevoflurane, suggesting that H_2_O_2_ is involved in the modulation of sevoflurane-induced ferroptosis. Moreover, sevoflurane upregulated TFR and TF and downregulated FPN, and these effects were attenuated when intracellular H_2_O_2_ was decreased by pretreatment with GSH. Furthermore, we found that exogenous H_2_O_2_ caused lipid peroxidation and neuronal cell death, which were accompanied by an increase in ferrous iron and the upregulation of TFR and TF, as well as the downregulation of FPN. Therefore, H_2_O_2_ contributed to the sevoflurane-induced increase in iron by upregulating TFR and TF and downregulating FPN, which resulted in iron-dependent lipid peroxidation and resultant ferroptosis.

ATF3 is a stress response factor that belongs to the ATF/CREB transcription factor family, and its expression is rapidly induced by multiple types of cellular stresses, including ischemia and hypoxia, oxidative stress, and cell damage [51]; this factor regulates different cell fate decisions. ER stress has been widely acknowledged as a major source of superoxide and H_2_O_2_, which etiologically links cell death to various neurological disorders and ischemic injury [29–31, 52]. As a stress sensor, ATF3 can be activated by ER stress, and upon activation, ATF3 has been shown to improve intracellular H_2_O_2_ levels and subsequently participate in the regulation of various cell death consequences, such as apoptosis, autophagy, and ferroptosis [24, 26, 32, 53]. In this study, we observed that sevoflurane increased ATF3 expression in neuronal cells and the hippocampi of neonatal mice, which was paralleled by the upregulation of GRP78, PERK, and ATF4. In contrast, sevoflurane-induced ATF3 expression was significantly decreased when the upregulation of PERK and ATF4 was alleviated by the PERK inhibitor GSK2606414 and knockdown of ATF4, indicating that ATF3 expression after sevoflurane exposure was induced by ER stress through the PERK/ATF4-mediated pathway. It has been proven that the PERK pathway acts as an upstream regulator of ATF3 during ER stress, which upregulates ATF3 and induces its translocation into the nucleus, resulting in retinal pigment epithelial cell ferroptosis [32]. Consistently, this study showed that knockout of ATF3 expression not only suppressed the sevoflurane-induced increase in H_2_O_2_ and neuronal death but also prevented increases in lipid peroxidation and ferrous iron, the upregulation of TF and TFR, and the downregulation of FPN in vitro and in vivo, suggesting that ATF3 promoted sevoflurane-induced neuronal ferroptosis by increasing intracellular H_2_O_2_. In addition, we found that neuronal death and the increases in H_2_O_2_, ferrous iron and lipid peroxidation, as well as the upregulation of TFR and TF and downregulation of FPN caused by sevoflurane, were significantly alleviated by GSK2606414 or ATF4 knockdown. Therefore, these results suggested that sevoflurane exposure induced neuronal cell ferroptosis in the developing brain, which was initiated by ATF3 activation through the ER stress-mediated PERK/ATF4 pathway and induced cell death caused by intracellular H_2_O_2_ accumulation and iron overload.

During the progression of ferroptosis, intracellular H_2_O_2_ can be increased when NADPH oxidase (NOX) is upregulated or catalase is downregulated [28]. NOX4, an NADPH oxidase, is mainly located in the endoplasmic reticulum and can produce superoxide and subsequently increase intracellular H_2_O_2_ in the presence of SOD [42, 54]. Catalase is a conjugated enzyme that uses iron porphyrin as its prosthetic group, has strong H_2_O_2_ scavenging activity, and can convert H_2_O_2_ reduction into oxygen and water [43]. Studies have shown that sevoflurane can activate NADPH oxidase and superoxide production, which in turn leads to lipid peroxidation damage and neuronal cell death in the developing brain [49, 54]. Similarly, our results showed that sevoflurane exposure increased intracellular superoxide and H_2_O_2_, which was accompanied by NOX4 upregulation and catalase downregulation, while inhibiting NOX4 with the specific inhibitor GKT137831 markedly prevented the sevoflurane-induced increases in superoxide and H_2_O_2_, indicating that NOX4 and catalase may mediate the sevoflurane-induced increase in H_2_O_2_. Emerging evidence links ER stress to an increase in intracellular H_2_O_2_, involving the activation of NOX4 or inhibition of catalase caused by ATF3 overexpression [55], and our results showed that ATF3 silencing in neuronal cells and ATF3 gene knockout in mice not only inhibited the sevoflurane-induced increase in H_2_O_2_ but also prevented the upregulation of NOX4 and downregulation of catalase. Therefore, ATF3 promoted the increase in intracellular H_2_O_2_ induced by sevoflurane by upregulating NOX4 and downregulating catalase.

GPX4 is a primary antioxidant enzyme that is part of an intracellular antioxidant system that senses oxidative stress damage, scavenges H_2_O_2_, and inhibits lipid peroxidation by consuming intracellular GSH [44]. As the functional subunit, the light chain encoded by SLC7A11 is crucial for the activity of amino acid antiporter system Xc − that involves the exchange of extracellular cystine and intracellular glutamate and is responsible for cysteine production and the subsequent synthesis of GSH [32, 45]. Studies have shown that suppressing GPX4 by RSL3 or inhibiting system Xc − with erastin could deplete cysteine and GSH and increase H_2_O_2_, leading to lipid peroxidation and ferroptosis [44, 49, 56]. Consistently, we observed that sevoflurane exposure could deplete cysteine and GSH by suppressing GPX4 and SLC7A11, which resulted in intracellular H_2_O_2_ production, suggesting that GPX4 and SLC7A11 mediated the sevoflurane-induced increase in H_2_O_2_. Increasing evidence suggests that ATF3 has a vital role in the regulation of cell death, given that it can suppress or activate transcription based on cell stress decisions [32]. Previous studies proved that ATF3 could repress SLC7A11 transcription by binding to the SLC7A11 promoter to suppress system Xc − , subsequently promoting ferroptosis caused by erastin [32, 57]. It was reported that ATF3 activation was associated with the inhibition of system Xc − , which resulted in an increase in intracellular H_2_O_2_ and ferroptosis in glioma cells [28]. Consistently, the present study showed that knockout of ATF3 expression not only inhibited the sevoflurane-induced downregulation of GPX4 and SLC7A11 but also mitigated the increase in H_2_O_2_ and depletion of cysteine and GSH in vitro and in vivo. Thus, ATF3 promoted the sevoflurane-induced accumulation of intracellular H_2_O_2_ by downregulating GPX4 and SLC7A11.

An increasing number of studies have proven that sevoflurane exposure can cause persistent cognitive impairment in neonatal rodents and nonhuman primates, which is closely related to hippocampal damage induced by various pathological processes, such as neuroinflammation and neuronal apoptosis [3–5, 11, 37, 58]. The present study proved that iron-mediated hippocampal neuronal damage was associated with memory impairment caused by repeated sevoflurane exposure in neonatal mice. We found that the iron chelator deferiprone (DFP) not only suppressed the increase in iron and neuronal death in the hippocampus but also prevented spatial learning and memory impairments in neonatal mice after sevoflurane exposure. Furthermore, we observed that sevoflurane-induced hippocampal neuronal ferroptosis and spatial memory impairment in neonatal mice were significantly alleviated by pretreatment with GSK2606414 or ATF3 silencing. These data suggested that ER stress and ATF3 activation-mediated hippocampal neuronal ferroptosis were implicated in the cognitive impairment caused by sevoflurane in neonatal mice. This result was consistent with recent evidence that treatment with DFP or GSK2606414 markedly improved motor function and mitigated degenerative alterations in Parkinson’s disease and frontotemporal dementia [59, 60]

In summary, we demonstrated that ER stress-induced PERK/ATF4-mediated ATF3 activation contributed to sevoflurane-induced neonatal neuronal ferroptosis, which was induced by iron overload via an increase in intracellular H_2_O_2_. The present study provides important insights into the mechanisms of sevoflurane-induced developmental neurotoxicity and suggests a potential treatment strategy to protect against neuronal damage from ferroptosis that contributes to neuronal cell death when volatile anesthesia is used in young children and newborns.

### Supplementary Information

Below is the link to the electronic supplementary material.

Supplementary file1 (TIF 18903 KB)

## Data Availability

The datasets utilized and/or analyzed during the present investigation are available from the corresponding author upon reasonable request.
