# Supplementary figures and images for: Sevoflurane Exposure Induces Neuronal Cell Ferroptosis Initiated by Increase of Intracellular Hydrogen Peroxide in the Developing Brain via ER Stress ATF3 Activation
Source: Mol Neurobiol. 2023 Oct 24;61(4):2313–35. doi: 10.1007/s12035-023-03695-z (PMC10972952; doi:10.1007/s12035-023-03695-z)

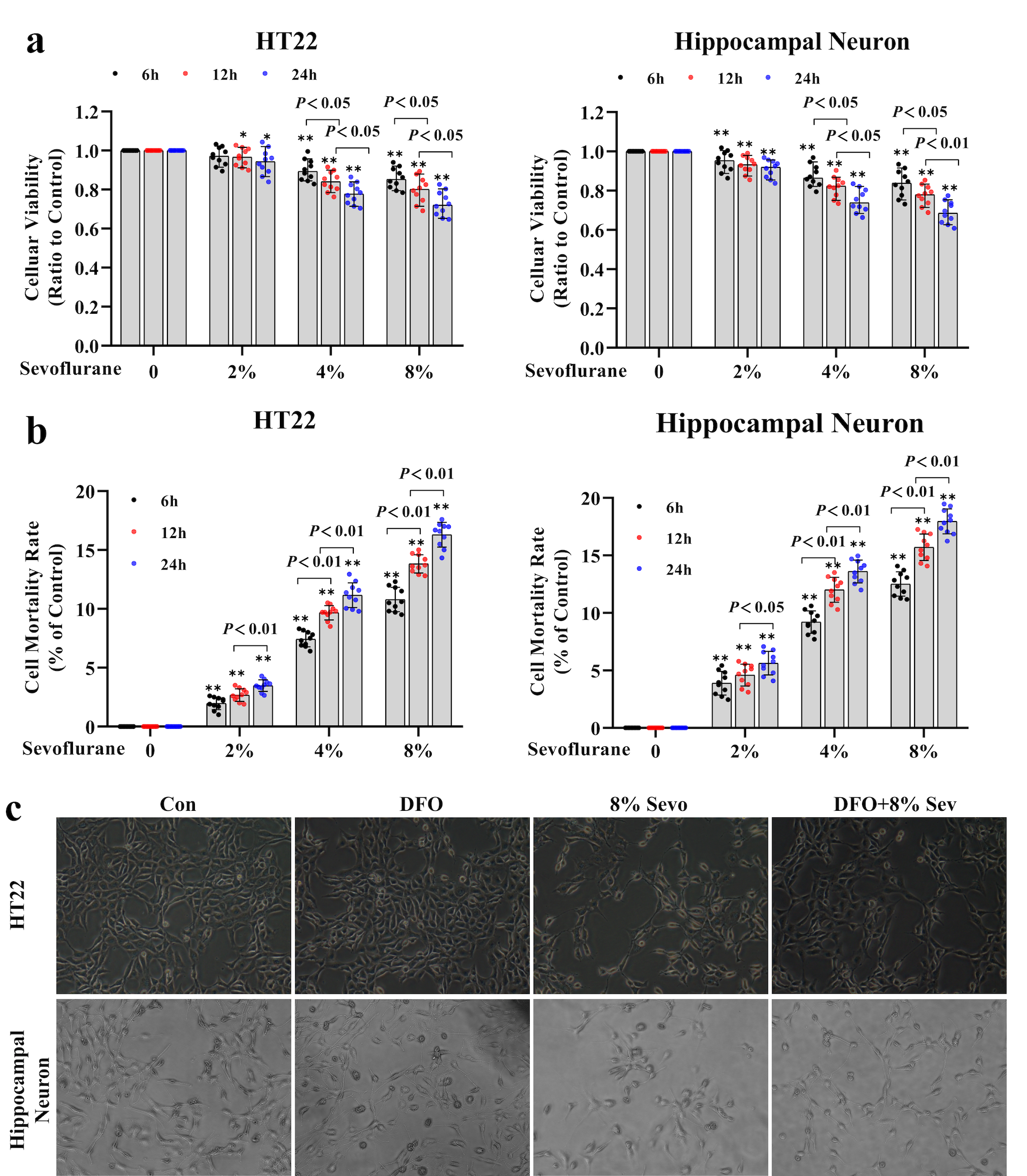

Supplement: Supplementary file 1 — Supplementary file1 (TIF 18903 KB) [file 12035_2023_3695_MOESM1_ESM.tif]
